# Clinical and haematological correlates of severe dengue using 2023 epidemic data: A multicentre tertiary hospital-based analysis from Dhaka, Bangladesh

**DOI:** 10.1371/journal.pntd.0013729

**Published:** 2026-07-30

**Authors:** M. Pear Hossain, Rudbar Mahmood, Farzana Sultana Bari, Afrin Naher Kana, Bibek Ahamed, Munia Afroza Shanta, Sadia Afrin, Musleha Akter, Tahmina Akter Anika, Mehejabin Nurunnahar, Sayeda Jahin Tasnim, Abu Bakkar Siddique, Linyan Li, Sheikh Taslim Ali

**Affiliations:** 1 WHO Collaborating Centre for Infectious Disease Epidemiology and Control, School of Public Health, Li Ka Shing Faculty of Medicine, The University of Hong Kong, Hong Kong Island, Hong Kong Special Administrative Region, Hong Kong, China; 2 Laboratory of Data Discovery for Health Limited, Hong Kong Science Park, New Territories, Hong Kong Special Administrative Region, Hong Kong, China; 3 Department of Public health, North South University, Dhaka, Bangladesh; 4 Department of Food Engineering and Nutrition Science, State University of Bangladesh, Dhaka, Bangladesh; 5 Department of Statistics, Jahangirnagar University, Dhaka, Bangladesh; 6 Infectious Diseases Division, International Centre for Diarrhoeal Disease Research, Dhaka, Bangladesh; 7 Maternal and Child Health Division, International Centre for Diarrhoeal Disease Research, Dhaka, Bangladesh; 8 Institute of Epidemiology, Disease Control and Research, Dhaka, Bangladesh; 9 Department of statistics, Eden Mohila College, Dhaka, Bangladesh; 10 Department of Data Science, City University of Hong Kong, Hong Kong Special Administrative Region, Kowloon, China; 11 Department of Infectious Diseases and Public Health, Jockey Club College of Veterinary Medicine and Life Sciences, City University of Hong Kong, Hong Kong Special Administrative Region, Kowloon, China; University of Dhaka, BANGLADESH

## Abstract

**Background:**

Dengue fever, caused by the dengue virus (DENV), remains a major public health threat in tropical and subtropical regions. Bangladesh recoded its largest dengue outbreak in 2023. Understanding factors associated with dengue severity is essential to improve clinical triage and outcome.

**Methods:**

We conducted a hospital-based study of 466 laboratory-confirmed dengue cases admitted to four tertiary hospitals in Dhaka during the peak epidemic period (September 1 to October 31, 2023). Demographic, clinical, and laboratory data were collected using a semi-structured questionnaire. Cases were categorized based on severity, and multivariable models were used to identify factors associated with severe dengue.

**Results:**

Severe dengue was independently associated with older age (adjusted OR [aOR] 2.381, 95% confidence interval [CI] 2.378–2.385) and higher pulse rate (aOR 1.035, 95% CI 1.034–1.037). After adjustment, higher BMI showed a protective association with severe dengue (aOR 0.094, 95% CI 0.094–0.094,). Higher platelet count (aOR 0.834, 95% CI 0.832–0.835), hematocrit (aOR 0.010, 95% CI 0.010–0.010), white blood cell (WBC) count (aOR 0.594, 95% CI 0.593–0.595), and body temperature (aOR 0.543, 95% CI 0.542–0.544) were also associated with reduced odds of severe outcomes. Delays in seeking care were associated with increased severity (*p* = 0.007). Symptom-based markers, such as abdominal pain (*p* < 0.001) and respiratory distress (*p* = 0.013), were strong predictors of severe dengue.

**Conclusions:**

Risk of severe dengue was driven by demographic and clinical features, and was exacerbated by delayed presentation. The observed association involving BMI warrants further study. These findings may support targeted, timely clinical management during outbreaks.

## Author summary

Dengue poses a significant public health challenge in Bangladesh. To better understand the epidemiological and clinical characteristics of dengue in Dhaka city, we conducted a study on 466 patients admitted to four distinct tertiary hospitals during the 2023 outbreak. Dengue cases were identified through serological testing for the presence of the NS1 antigen or IgM antibody. Our analysis revealed that older age, elevated diastolic blood pressure, increased pulse rate, and lower body mass index, as well as reduced platelet count, hematocrit, and white blood cell count, were significantly associated with severe dengue outcomes. Furthermore, delays in seeking medical care were strongly correlated with increased disease severity, highlighting the critical importance of timely intervention. Symptoms such as abdominal pain and respiratory distress emerged as strong indicators of severe dengue. These findings emphasize the complex interplay of various factors in determining dengue severity and underscore the need for context-specific public health strategies. By identifying these factors, healthcare providers can enhance the management of severe dengue cases and reduce the impact of future outbreaks.

## Introduction

**Fig 1:**
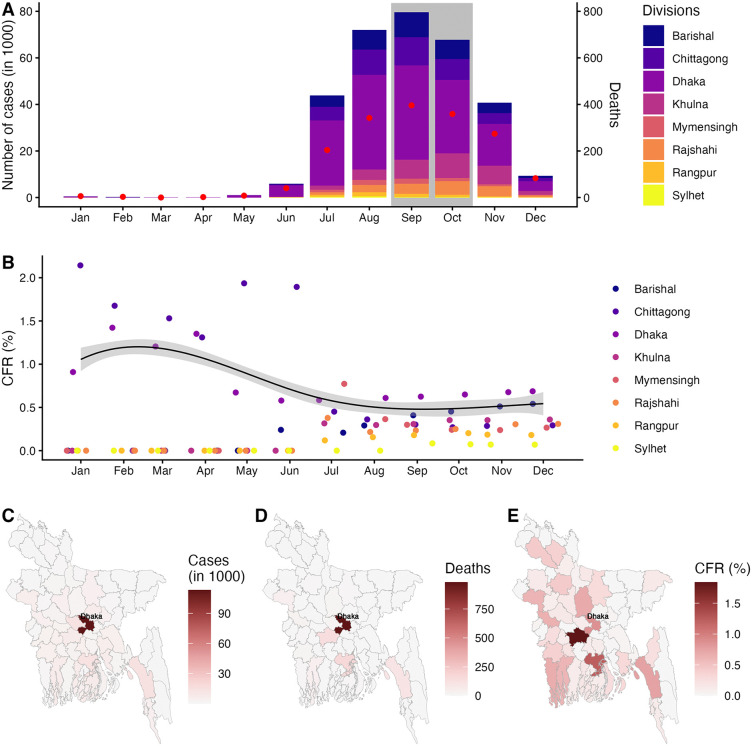
Dynamics of dengue outbreak in Bangladesh, 2023. **(A)** Monthly dengue cases across the eight primary administrative regions (divisions) of Bangladesh. The red dots in each month indicate the total number of deaths in Bangladesh for 2023. The grey shaded region indicates the period during which data were collected from individual patients upon hospital admission. **(B)** Case Fatality Rate (CFR, in %) by month and division, calculated as the percentage of cumulative deaths in each month divided by cumulative cases in that month. The solid line represents the overall CFR with 95% CI (grey shade) for Bangladesh across the months. **(C)** Spatial distribution of dengue cases across districts in Bangladesh, highlighting Dhaka district (second-level administrative region) as the area reporting the highest number of cases. **(D)** Spatial distribution of dengue-related deaths, with Dhaka district again showing the highest numbers. **(E)** Spatial distribution of CFR (%) across districts, with Faridpur district exhibiting the highest CFR, located adjacent to Dhaka district. Maps were generated using the gadm function from the geodata R package, using administrative boundary data obtained from GADM. The GADM licensing allows republication for academic and non-commercial use, including use of maps in figures for published research articles under open licenses (e.g., CC-BY), subject to GADM’s terms; licensing details are available at https://gadm.org/license.html.

Dengue fever, a mosquito-borne viral disease caused by the dengue virus (DENV), remains a major global public health threat, particularly in tropical and subtropical regions [1]. With an estimated 390 million infections annually, dengue imposes a substantial burden on healthcare systems, especially in endemic areas such as South and Southeast Asia [2]. In Bangladesh, dengue has emerged as a major health concern, with recurrent outbreaks and increasing severity in recent years. In 2023, the dengue outbreak was particularly catastrophic, with 321179 reported cases and 1705 deaths (Fig 1A & 1B), highlighting the urgent need for a deeper understanding of factors driving severe disease progression [3].

Severe dengue, as defined by the World Health Organization (WHO) 2009 guidelines, is characterized by plasma leakage, severe hemorrhage, and organ impairment, often leading to life-threatening complications such as shock and multi-organ failure [4]. While the majority of dengue cases are mild or asymptomatic, early identifying of severity predictors is vital for timely interventions and resource allocation. Established risk factors include older age, comorbidities (e.g., diabetes, mellitus and hypertension), and delays in seeking medical care [5–9]. However, the role of body mass index (BMI) in dengue severity remains controversial, with some studies suggesting obesity as a risk factor and others reporting no significant association [8,10–12]. This discrepancy underscores the need for population-specific studies to clarify the relationship between metabolic health and dengue outcomes.

Dhaka City emerged as the epicentre of dengue outbreak in 2023, contributing 34% of national cases (110008) and 57% of dengue-related deaths (980) in Bangladesh (Fig 1C-1E). This high-burden setting provided a unique opportunity to investigate various risk factors associated with disease severity. Despite the high burden of dengue in this region, limited data exist on the demographic, clinical, and laboratory profiles associated with severe outcomes, particularly during epidemic periods. Understanding these factors is essential for improving clinical management, guiding public health interventions, and reducing mortality in resource-limited settings.

This study aimed to identify key risk factors of severe dengue using data collected from patients upon hospital admission during the 2023 outbreak in Dhaka City. By analyzing demographic characteristics, clinical timelines, comorbidities, and laboratory parameters, we sought to provide insights into the factors driving disease severity and inform targeted strategies for patient care and outbreak response. Our findings contribute to the epidemiological evidence on dengue risk factors and highlight the importance of target-specific outbreak mitigation strategies to address context-specific dengue transmission dynamics.

## Methods

### Ethical declaration

The Institutional Ethical Approval Committee of Primeasia University, Bangladesh (PAU/IEAC/23/123) reviewed and approved the study. Prior to conducting the interviews, written consent was obtained from each patient. At the time of obtaining ethical approval, Farzana Sultana Bari held a prior faculty affiliation with Primeasia University, which facilitated the ethical review process.

### Study design, setting and participants

This study employed a descriptive observational design [13] and was conducted across four tertiary hospitals in Dhaka City, Bangladesh. The participating hospitals included three branches of Islami Bank Hospital (located in Kakrail, Mugda, and Mirpur) and Enam Medical College Hospital in Savar. Data collection took place from September 1 to October 31, 2023, during the peak dengue epidemic in Dhaka. All dengue patients admitted to the designated wards during this period were considered eligible. Patients were included if they had laboratory-confirmed dengue infection, defined by a positive IgM antibody test and/or NS1 antigen test. Written consent was obtained from each hospital administration to access the dengue wards. Additionally, informed consent was obtained from all patients (or from parents/guardians for minors under 18 years) prior to interviews. A total of 466 laboratory-confirmed dengue patients were enrolled in the study.

In each hospital, two trained data collectors were responsible for data collection during the study period. They were present in the hospitals during daytime hours to gather data. For consenting patients, face-to-face interviews were conducted using a semi-structured questionnaire to collect demographic information (e.g., age, sex, occupation), medical history (e.g., previous health conditions, comorbidities, COVID-19 vaccination status), and clinical manifestations (e.g., symptoms at presentation and during illness). Laboratory data, including test results, disease severity, repeated hematological measurements, vital signs, and liver function tests, were extracted from medical records. Patients were followed throughout their hospitalization, with clinical and laboratory data updated and recorded until discharge.

### Defining dengue cases and other variables

Dengue cases were classified according to the WHO guidelines into dengue (with or without warning signs) and severe dengue [4]. Suspected cases were defined as patients presenting with fever (≥100.4°F) lasting 2–7 days accompanied by at least two of the following: headache, retro-orbital pain, myalgia, arthralgia, rash, hemorrhagic manifestations, or leukopenia. Laboratory confirmation was obtained through NS1 antigen testing for samples collected within the first five days of fever onset or IgM antibody testing for samples collected after five days [14]. Severe dengue was defined by the presence of at least one of the following criteria: (a) *Severe plasma leakage*, determined by significant fluid buildup in the body, which can lead to complications such as swelling in the abdomen or lungs. This condition may be accompanied by an increased heart rate, low blood pressure and/or a narrow difference between systolic and diastolic blood pressure, suggesting critical drop in blood volume. (b) *Severe hemorrhage*, involves serious bleeding that can pose life-threatening risk. Symptoms may include vomiting blood, passing fresh blood in the stool, or having dark, tarry stools. These bleeding episodes are often associated with unstable blood pressure and a rapid drop in hemoglobin levels, further complicating the patient’s condition. (c) *Severe organ impairment*, signifies the dysfunction of vital organs and can manifest as confusion or altered consciousness, severe liver damage (indicated by significantly elevated levels of liver enzymes, ALT or AST), or inflammation of the heart muscle (myocarditis) [15]. Body mass index (BMI) is classified into three categories: underweight (<18.5), normal (18.5-24.9), and overweight (≥25). The length of hospital stay is the period from admission to discharge. Patients with previous medical conditions such as COPD, Asthma, Hypertension, Diabetes mellitus, Heart disease, or other comorbidities are considered to have comorbidities.

### Statistical analysis

The included patients were categorized into two groups based on the case severity (i.e., severe and non-severe dengue cases), and suitable statistical tests were performed to compare. Categorical variables were compared using the Chi-squared test, while continuous variables were compared using the parametric t-test and non-parametric Manne-Whitney U test upon validation of normality assumptions. A 2-tailed p-value less than 0.05 was considered statistically significant.

In order to check the dynamics of severity profiles in different clinical phases (febrile, critical and recovery phases), clinical and laboratory variables were observed and compared overtime with reference to the symptom onset. This comparison was made between the severe and non-severe dengue groups using Manne-Whitney U test for each day and by group overall.

We further assessed risk factors of disease severity using two modeling approaches. First, to account for the longitudinal structure of the data, we fitted generalized linear mixed-effects models (GLMMs) with a binomial outcome and logit link function to clinical and laboratory variables measured repeatedly during hospitalization. The GLMM framework allowed us to incorporate both fixed effects for covariates of interest (e.g., baseline risk factors and time-varying clinical/laboratory measurements) and random effects to model sources of correlation and heterogeneity. Specifically, patient-level random intercepts were included to capture within-patient correlation over time, while hospital-level random effects were used to reflect inter-hospital variability in baseline severity risk and clinical practice patterns. Odds ratios and 95% confidence interval (CI) derived from the model coefficients were used to quantify the association between each predictor and dengue severity. Second, logistic regression was used for variables measured at the admission. In addition to this, another model was used to assess the association between clinical factors measured at admission and blood transfusion requirements, which we used as an indicator of increased dengue severity. All models were adjusted for demographic characteristics including age and BMI. Missing data were excluded from the analysis.

## Results

In this study, we analyzed data collected during the peak of the 2023 outbreak (September 1 to October 31, 2023) in Dhaka, Bangladesh. The dataset comprised demographic, clinical, and laboratory information of 466 patients admitted to four hospitals in Dhaka (*details in Methods section*). After cleaning the data to confirm NS1 or IgM positivity, 449 patients were retained for the main analysis. Among these, 66/449 (14.7%) met criteria for severe dengue and 383/449 (85.3%) for non-severe dengue. Dengue hemorrhagic fever was identified in 57/449 (12.7%) patients, and severe organ impairment in 9/449 (2.0%).

### Demographic and clinical characteristics

Cases of severe dengue found to be associated with a higher mean age compared to non-severe cases (39 ± 18 vs. 32 ± 18 years; *p* = 0.006), with a significant age-dependent trend in disease severity. The proportion of severe cases increased progressively across age groups, reaching 24% (9/38) among individuals aged >60 years, followed by 18% (17/93) in those aged 41–60 years, 15% (34/224) in the 19–40 years, and 6% (6/94) in individuals aged ≤18 years (*p* = 0.035) (Table A in S1 Text). A significant higher prevalence was found for overweight individuals (BMI ≥ 25 kg/m²: 23% of severe cases vs. 13% in normal BMI and 3.2% in underweight; *p* = 0.022). Severe cases also exhibited longer mean onset-to-admission times (4.71 ± 1.88 days) than non-severe cases (3.99 ± 2.17 days; *p* = 0.003). Similarly, mean recovery times with reference to symptom onset were significantly longer for severe cases (8.72 ± 2.31 days) versus non-severe cases (7.68 ± 2.48 days; *p* < 0.001).

### Comorbidities and disease severity

The presence of any comorbidity significantly increased the likelihood of severe dengue outcomes (20% vs. 13% in non-severe cases; p = 0.039). While diabetes mellitus (22% vs. 14%; p = 0.10) and hypertension (20% vs. 14%; p = 0.2) demonstrated elevated severity rates, these associations were not statistically significant. No significant associations were observed for COPD/asthma (p = 0.502), heart disease (p = 0.548), or other comorbidities (p = 0.3) (Table B in S1 Text).

### Symptomatic markers of severity

Abdominal pain (p < 0.001) and itchiness (p < 0.001) were strongly associated with severe outcomes. Respiratory distress (p = 0.013), joint pain (p = 0.026), and headache (p = 0.046) also showed significant associations with severe dengue. In contrast, fever (p = 0.5), rash (p = 0.4), and vomiting (p = 0.3) were found to be insignificant associations with severity. (Table C in S1 Text).

### Temporal profile of clinical factors during time-since-symptom-onsets

**Fig 2:**
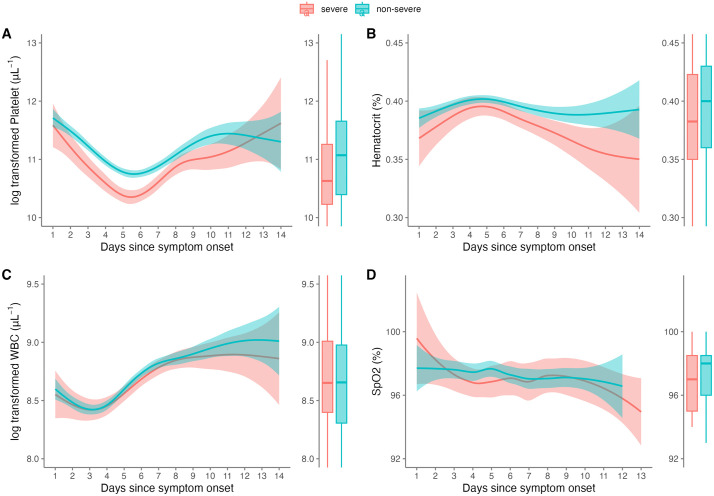
Dynamics of hematological profile among dengue patients admitted to the hospital. **(A)** Platelet counts measured in per microlitre blood (*µL*^−1^), **(B)** haematocrit measured in (%), **(C)** white blood cell count measured in per microlitre blood (*µL*^−1^), and **(D)** oxygen saturation (%). In each panel, lines and shaded regions indicate the fitted line obtained using generalized additive models, along with 95% confidence intervals (CIs). The boxplot in each panel represents the distributions of hematological profile categorized by severity levels. Asterisks denotes the significant p-values from the Mann-Whitney U test comparing the hematological profiles between two groups of dengue patients: severe vs non-severe.

**Fig 3:**
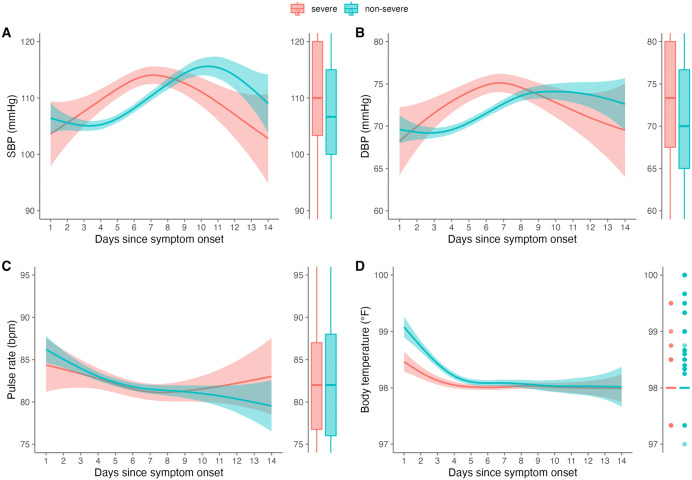
Dynamics of vital signs monitoring among dengue patients admitted to the hospital. **(A)** Systolic blood pressure measured in mmHg, **(B)** diastolic blood pressure measured in mmHg, **(C)** pulse rate measured in bpm (beats per minute), and **(D)** body temperature (℉). In each panel, lines and shaded regions indicate the fitted line obtained using generalized additive models, along with 95% confidence intervals (CIs). The boxplot in each panel represents the distributions of vital signs categorized by severity levels. Asterisks denotes the significant p-values from the Mann-Whitney U test comparing vital signs between two groups of dengue patients: severe vs non-severe.

**Fig 4:**
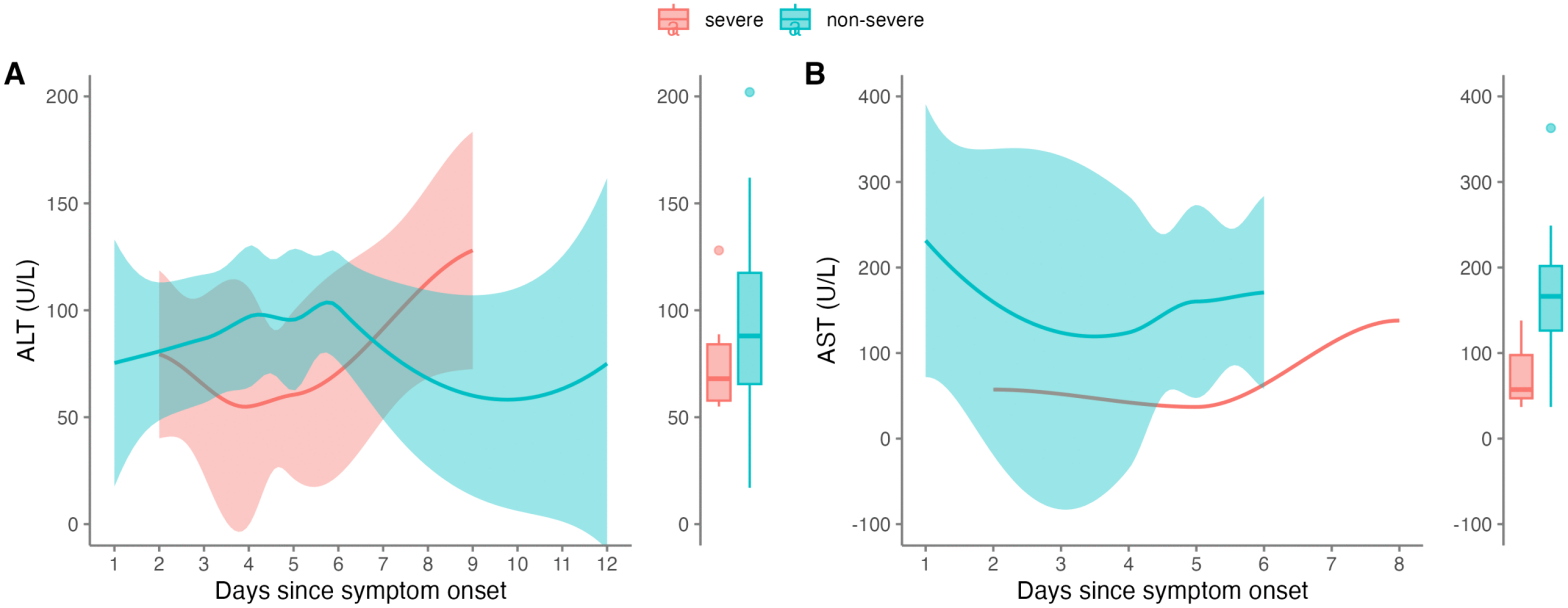
Dynamics of liver function tests among dengue patients admitted to the hospital. **(A)** ALT (alanine aminotransferase) and **(B)** AST (aspartate aminotransferase) measured in units per liter (U/L). In each panel, lines and shaded regions indicate the fitted line obtained using generalized additive models, along with 95% confidence intervals (CIs). The boxplot in each panel represents the distributions of liver function tests categorized by severity levels. Asterisks denotes the significant p-value from the Mann-Whitney U test comparing liver function tests over time between two groups of dengue patients: severe vs non-severe.

Significant differences were observed in log-transformed platelet levels between severe and non-severe dengue cases with clear differences during 3–7 days of post-symptom onset (Fig 2A). The severe dengue cases exhibited lower platelet levels during this period. The lowest log-transformed platelet levels were observed around day 6 of post-symptom onset in both groups. Hematocrit levels also showed significant differences between severe and non-severe dengue cases (Fig 2B). Specifically, hematocrit percentages were lower in the severe dengue group between 9 and 11 days post-symptom onset. Both severity groups showed a progressive decline in hematocrit between days 5 and 7 post-onset. No statistically significant differences were observed in WBC count trajectories or SpO2 measurements between severity groups. The vital signs monitoring revealed significantly elevated SBP and DBP in severe dengue patients compared to non-severe cases within the first week, specifically on days 4–7 post-symptom onset (Fig 3A & 3B). However, SBP was lower among the severe group between 10 and 12 days post-symptom onset. Peak SBP occurred on day 7 in the severe group and on day 11 in the non-severe group. Liver function parameters showed no significant variation between severe and non-severe dengue cases (Fig 4).

### Factors associated with dengue severity

Using a generalized linear mixed-effects model with a binomial link to account for repeated measurements within patients and clustering by hospital, we identified several factors associated with dengue severity (Table 1). The model revealed that older age (Adjusted OR [aOR]: 2.381, 95% CI 2.378–2.385, p < 0.001) and higher pulse rate (aOR 1.035, 95% CI 1.034–1.037, p < 0.001) were strong indicators of severe dengue, implying that each incremental increase in age and heart rate significantly increased the likelihood of severity. Several factors showed protective effects against severe dengue. Notably, higher BMI was associated with increased risk in unadjusted analysis; however, after adjusting for confounding variables, higher BMI was linked to a substantially lower odds of severe dengue (aOR 0.094, 95% CI 0.094–0.094, p < 0.001). Similarly, higher platelet counts (aOR 0.834, 95% CI 0.832–0.835, p < 0.001), hematocrit levels (aOR 0.010, 95% CI 0.010–0.010, p < 0.001), white blood cell counts (aOR 0.594, 95% CI 0.593–0.595, p < 0.001), and body temperature (aOR 0.543, 95% CI 0.542–0.544, p < 0.001) were all associated with a lower likelihood of severe dengue. Regarding blood pressure, systolic pressure showed a modest protective effect (aOR 0.992, 95% CI 0.991–0.994, p < 0.001), indicating that higher systolic values slightly decrease the risk of severe dengue. Conversely, diastolic pressure was associated with a slight increase in risk (aOR 1.011, 95% CI 1.009–1.012, p < 0.001), suggesting that higher diastolic pressure modestly elevates the likelihood of severity. The second model focused on factors associated with the requirement for blood transfusion, a clinical indicator of severe disease. This analysis revealed that lower platelet counts at admission were significantly associated with a higher likelihood of blood transfusion (Tables D-E in S1 Text), highlighting the importance of initial platelet levels as a predictor of severe clinical outcomes.

**Table 1:** Factors affecting dengue severity. For clinical factors with repeated measurements during hospital stay, a generalized linear mixed-effects model with a binomial link function was used, with individual patients and hospitals included as random effects.

Factors	uOR (95% CI)	p-value	aOR (95% CI)	p-value
Age (year)	1.568 (1.567 - 1.570)	0.0000	2.381 (2.378 - 2.385)	<0.001
BMI (kg/m^2^)	1.392 (1.185 - 1.634)	0.0001	0.094 (0.094 - 0.094)	<0.001
SBP (mmHg)	1.001 (0.972 - 1.031)	0.9483	0.992 (0.991 - 0.994)	<0.001
DBP (mmHg)	1.001 (0.969 - 1.034)	0.9458	1.011 (1.009 - 1.012)	<0.001
Pulse rate (bpm)	1.000 (0.961 - 1.039)	0.9811	1.035 (1.034 - 1.037)	<0.001
Body temperature (°F)	0.989 (0.650 - 1.507)	0.9598	0.543 (0.542 - 0.544)	<0.001
Platelet (*µL* ^− 1^)	0.970 (0.611 - 1.541)	0.8982	0.834 (0.832 - 0.835)	<0.001
Hematocrit (%)	0.998 (0.334 - 2.978)	0.9966	0.010 (0.010 - 0.010)	<0.001
WBC (*µL* ^− 1^)	1.028 (0.260 - 4.058)	0.9687	0.594 (0.593 - 0.595)	<0.001

Odds ratios (OR), 95% confidence intervals (CI), and p-values are reported for both univariate (left) and multivariate (right) models. uOR represents unadjusted OR and aOR represent adjusted OR. Platelet count and WBC were log-transformed in the model. The final model included 1,387 observations from 241 patients across 4 hospitals. Missing data were excluded from the analysis. BMI: body mass index, SBP: systolic blood pressure, DBP: diastolic blood pressure, WBC: white blood cell.

## Discussion

The 2023 dengue outbreak was the most devastating ever recorded in Bangladesh, resulting in an exceptionally high number of cases of morbidity and mortality. This surge may have been influenced by a shift in the predominant serotype, from DENV-3 in 2019 to DENV-2 in 2023 [16]. In this study, we identified several critical factors associated with severe dengue progression. Older age, reduced platelet counts, and lower body mass index emerged as independent risk factors for disease severity. Clinical timelines, including delays between symptom onset and hospital admission, prolonged hospital stay, and extended time from onset to recovery, were significantly linked to severe outcomes. Additionally, the presence of comorbidities and specific clinical manifestations including headache, abdominal pain, joint pain, respiratory distress, and itchiness were strongly associated with the outcomes of disease severity.

The WHO 2009 guidelines classify dengue progression into three clinical phases—febrile (days 1–3 post-onset), critical (days 3–6), and recovery (>6 days)—with distinct hematological dynamics, including platelet count decline and hematocrit elevation [4]. During the critical phase, platelet counts reach their nadir concurrent with peak hematocrit levels, a hallmark of plasma leakage and thrombocytopenia [17]. Our findings align with these established patterns, showing that log-transformed platelet count reached their lowest point on day 6 post-symptom onset in both groups (Fig 2). Additionally, our results demonstrate that lower platelet counts are strongly associated with progression to severe dengue, reinforcing their role as a key WHO warning sign [4]. This corroborates observational studies in diverse healthcare settings, where thrombocytopenia consistently predicts severe outcomes [18,19]. Conversely, in our study, higher hematocrit levels were associated with a lower likelihood of severe dengue, which contrasts with prior research indicating that elevated hematocrit is a marker of disease severity in dengue infection [20].

Our findings, which indicate an association between lower BMI and more severe dengue outcomes, contrast with studies reporting obesity as a risk factor for severe manifestations (Table 1). For example, a recent systematic review reported that patient with obesity had a 50% higher likelihood of developing severe dengue (OR = 1.50, 95% CI: 1.15–1.97) [11]. In Thailand, overweight pediatric patients (BMI ≥ 25) showed a higher prevalence of severe plasma leakage compared to those with mild leakage (45.5% vs. 18.8%) [21]. This discrepancy may reflect population-specific differences in metabolic health, age-related immune responses, or comorbidities. Although obesity has been hypothesized to exacerbate dengue severity through mechanisms such as chronic inflammation and endothelial dysfunction [11], our results suggest that undernutrition or metabolic alterations associated with lower BMI may also contribute to adverse outcomes. Possible pathways include impaired immune competence and delayed recovery. Consistence with this, atypical clinical presentations, such as encephalopathy and fluid overload, have been reported among overweight pediatric cohorts [22,23], emphasizing the need for context-specific clinical vigilance. However, evidence from other settings where obesity was not associated with severe dengue or mortality [12] highlights the complexity of BMI as a prognostic indicator. These inconsistencies may arise from variations in study design, regional differences in circulating dengue serotypes, or heterogeneity in how obesity and BMI categories are defined across studies.

The elevated risk of severe dengue in older adults may reflect a higher prevalence of comorbidities in this population (Table 1). Although our study did not identify statistically significant associations with specific comorbidities, possibly due to limited sample size or heterogeneous profiles, existing literature highlights diabetes mellitus, hypertension, and chronic kidney disease as critical contributors to severe dengue in older individuals [6,24]. These conditions may exacerbate severity through mechanisms such as impaired immune responses (e.g., immunosenescence) and endothelial dysfunction, which can enhance plasma leakage and thrombocytopenia during the critical phase of infection. For instance, diabetes-associated hyperglycemia may disrupt platelet function, worsening thrombocytopenia, while hypertension could accelerate vascular permeability, explaining the higher proportion of severe dengue cases observed in our findings.

The mean onset-to-admission times and mean recovery times since symptom onset were found to be significantly longer for severe cases (Table A in S1 Text). The association between clinical timelines and severe dengue outcomes is well-documented in the literature [25–27]. Delays in seeking medical attention after symptom onset can significantly worsen prognosis. A systematic review highlighted that prolonged intervals between symptom onset and hospital admission are linked to increased severity of the disease, as timely intervention is crucial for managing complications such as hemorrhagic manifestations and shock [27]. Moreover, the duration of hospital stay serves as an important indicator of severity. Patients with severe dengue often require longer hospital stays due to the need for intensive monitoring and management of complications [26]. This extended hospital stay not only reflects disease severity but also contributes to increased healthcare costs and resource utilization [26]. Furthermore, the time from symptom onset to recovery is critical; longer recovery times are associated with more severe disease trajectories, as those with significant delays often have underlying complications necessitating prolonged medical care [25]. This emphasizes the need for healthcare systems to enhance awareness of dengue symptoms and improve access to timely medical care to mitigate the risk of severe outcomes.

### Limitations

The study used data collected from patients upon hospital admission, providing precise individual level information on laboratory-confirmed dengue cases admitted to four tertiary hospitals in Dhaka, one of the most densely populated cities in the world. We identified significant critical factors associated with disease severity outcome for dengue. However, this study has several limitations that should be considered when interpreting the findings.

***First****,* the study was conducted in four tertiary hospitals in Dhaka City during the peak of the 2023 dengue outbreak. As these facilities primarily manage patients with more severe illness, this focus may introduce selection bias, potentially overrepresenting severe cases compared to community or primary care settings. This could limit the generalizability of our findings to other regions, healthcare levels, or non-epidemic periods. ***Second****,* the sample size, while substantial, may have been insufficient to detect statistically significant associations for certain comorbidities and symptoms, particularly those with low prevalence. ***Three****,* the reliance on hospital records and patient interviews for data collection may introduce recall bias, especially for variables such as symptom onset and duration. Inaccurate recall could affect the precision of timing-related variables and weaken associations with outcomes. ***Fourth****,* some variables, including repeated laboratory measurements during hospitalization, had missing data due to variations in clinical practice and patient availability. This may have reduced statistical power or introduced bias if missingness was related to disease severity or treatment decisions. ***Fifth****,* clinical management decisions, such as the timing and type of treatment administered, could have acted as confounders, influencing both laboratory values and outcomes. Since treatment facilities were not same across hospitals, variations in management may have affected the associations observed. ***Finally,*** the classification of BMI and comorbidities was based on self-reported or hospital-recorded data, which may not always be accurate.

## Conclusion

This study identified key demographic and clinical factors associated with severe dengue in the 2023 Dhaka epidemic. Older age was consistently and strongly linked to severe disease, even after adjusting for other factors. In the multivariable model, lower BMI emerged as a significant risk factor, suggesting that undernutrition or altered metabolic status may contribute to poorer outcomes. Higher DBP and pulse rate were independently associated with increased odds of severe dengue, while higher SBP, body temperature, platelet count, hematocrit, and WBC count were associated with lower odds, indicating potential protective or compensatory physiological responses. Delays in hospital admission, prolonged hospitalization, and extended recovery times were also strongly linked to severe outcomes, emphasizing the importance of timely medical intervention. The presence of comorbidities and specific symptoms, such as abdominal pain, respiratory distress, and itchiness, further underscored the complexity of dengue progression.

## Supporting information

S1 Text Supplementary information of Clinical and haematological correlates of severe dengue using 2023 epidemic data: a multicentre tertiary hospital-based analysis from Dhaka, Bangladesh. **Table A**. Associations between demographic characteristics, clinical timelines, and dengue severity in hospitalized patients during the 2023 Dhaka outbreak. **Table B.** Comparing the comorbidities between severe and non-severe dengue cases. **Table C.** Comparing the clinical manifestation between severe and non-severe dengue cases. **Table D.** Factors affecting dengue severity. For clinical factors measured at the time of admission, we conducted an additional analysis using a logistic regression model. Odds ratios (OR), 95% confidence intervals (CI), and p-values are reported for both univariate (left) and multivariate (right) models. Platelet count and white blood cell (WBC) count were log-transformed in the model. **Table E.** Factors affecting blood transfusion requirements of dengue cases. For clinical factors measured at the time of admission, we conducted an additional analysis using a logistic regression model. Odds ratios (OR), 95% confidence intervals (CI), and p-values are reported for both univariate (left) and multivariate (right) models. Platelet count and white blood cell (WBC) count were log-transformed in the model.
(DOCX)
