# Supplementary material for: Clinical and haematological correlates of severe dengue using 2023 epidemic data: A multicentre tertiary hospital-based analysis from Dhaka, Bangladesh
Source: PLoS Negl Trop Dis. 2026 Jul 30;20(7):e0013729. doi: 10.1371/journal.pntd.0013729 (PMC13440845; doi:10.1371/journal.pntd.0013729)
Supplement: Supplementary file 1 [file pntd.0013729.s001.docx]

**Supplementary -**

**Clinical and haematological correlates of severe dengue using 2023 epidemic data: a multicentre tertiary hospital-based analysis from Dhaka, Bangladesh**

**AUTHORS:**

M. Pear Hossain^1,2^, Rudbar Mahmood^3^, Farzana Sultana Bari^4^, Afrin Naher Kana^5,6^, Bibek Ahamed^5,7^, Munia Afroza Shanta^5,7^, Sadia Afrin^5,7^, Musleha Akter^5^, Tahmina Akter Anika^5^, Mehejabin Nurunnahar^8^, Sayeda Jahin Tasnim^9^, Abu Bakkar Siddique^7^, Linyan Li^10,11^, Sheikh Taslim Ali^1,2^

**AFFILIATIONS:**

^1^ WHO Collaborating Centre for Infectious Disease Epidemiology and Control, School of Public Health, Li Ka Shing Faculty of Medicine, The University of Hong Kong, Hong Kong, Hong Kong Special Administrative Region, China

^2^ Laboratory of Data Discovery for Health Limited, Hong Kong Science Park, New Territories, Hong Kong.

^3^ Department of Public health, North South University, Dhaka 1229, Bangladesh.

^4^ Department of Food Engineering and Nutrition Science, State University of Bangladesh, Narayanganj, Dhaka-1461, Bangladesh.

^5^ Department of Statistics, Jahangirnagar University, Dhaka, Bangladesh.

^6^ Infectious Diseases Division, International Centre for Diarrhoeal Disease Research, Bangladesh, Dhaka, Bangladesh.

^7^ Maternal and Child Health Division, International Centre for Diarrhoeal Disease Research, Bangladesh, Dhaka, Bangladesh.

^8^ Institute of Epidemiology, Disease Control and Research, Dhaka, Bangladesh.

^9^ Department of statistics, Eden Mohila College, Dhaka, Bangladesh.

^10^ Department of Data Science, City University of Hong Kong, Hong Kong, Hong Kong Special Administrative Region, China

^11^ Department of Infectious Diseases and Public Health, Jockey Club College of Veterinary Medicine and Life Sciences, City University of Hong Kong, Hong Kong, Hong Kong Special Administrative Region, China

Communicating author: [alist15@hku.hk](mailto:alist15@hku.hk) (STA), The University of Hong Kong, Hong Kong, Hong Kong Special Administrative Region, China

**List of tables**

**Table A.** Associations between demographic characteristics, clinical timelines, and dengue severity in hospitalized patients during the 2023 Dhaka outbreak.

| **Characteristic** | **Severe**  N = 66^1^ | **Non-severe**  N = 383^1^ | **p-value**^2^ |
| --- | --- | --- | --- |
| **Age group (years)** |  |  | **0.035** |
| $\leq$ 18 | 6 (6.4%) | 88 (94%) |  |
| 19-40 | 34 (15%) | 190 (85%) |  |
| 41-60 | 17 (18%) | 76 (82%) |  |
| 60+ | 9 (24%) | 29 (76%) |  |
| Mean (sd) | 39 (18) | 32 (18) | 0.006 |
| **Sex** |  |  | **0.921** |
| Female | 28 (15%) | 160 (85%) |  |
| Male | 38 (15%) | 223 (85%) |  |
| **BMI** |  |  | **0.022** |
| Underweight | 1 (3.2%) | 30 (97%) |  |
| Normal | 21 (13%) | 136 (87%) |  |
| Overweight | 19 (23%) | 64 (77%) |  |
| Mean (sd) | 24.5 (4.1) | 23.4 (4.9) | 0.083 |
| **Occupation** |  |  | **0.063** |
| Business | 12 (23%) | 41 (77%) |  |
| Housewife | 22 (20%) | 90 (80%) |  |
| Job holder | 10 (12%) | 72 (88%) |  |
| Student | 10 (8.5%) | 108 (92%) |  |
| Others | 5 (14%) | 31 (86%) |  |
| **Require blood/platelet transfusion** | | | **0.096** |
| Not required | 48 (14%) | 306 (86%) |  |
| Required | 16 (21%) | 60 (79%) |  |
| **Vaccinated for COVID-19** | |  | **0.076** |
| Unvaccinated | 5 (7.6%) | 61 (92%) |  |
| Vaccinated | 56 (16%) | 294 (84%) |  |
| **Vaccine doses** |  |  | **0.087** |
| none | 5 (7.6%) | 61 (92%) |  |
| single | 3 (25%) | 9 (75%) |  |
| full | 19 (13%) | 132 (87%) |  |
| booster | 34 (18%) | 153 (82%) |  |
| **Onset-to-admission (days)** | |  | **0.161** |
| $\leq$ median | 33 (51%) | 155 (41%) |  |
| $>$median | 32 (49%) | 219 (59%) |  |
| Mean (sd) | 4.71 (1.88) | 3.99 (2.17) | 0.003 |
| **Onset-to-recovery (days)** |  |  | **<0.001** |
| $\leq$median | 35 (54%) | 110 (30%) |  |
| $>$median | 30 (46%) | 256 (70%) |  |
| Mean (sd) | 8.72 (2.31) | 7.68 (2.48) | <0.001 |
| **Duration of hospital stay (days)** | |  | **0.334** |
| $\leq$median | 21 (32%) | 96 (26%) |  |
| $>$median | 45 (68%) | 272 (74%) |  |
| Mean (sd) | 4.02 (2.26) | 3.61 (1.69) | 0.405 |
| ^1^n (%); Mean (sd) | | | |
| ^2^Pearson's Chi-squared test; Wilcoxon rank sum test; Fisher's exact test | | | |

**Table B.** Comparing the comorbidities between severe and non-severe dengue cases.

| **Characteristic** | **Severe, N = 66^1^** | **Non-severe, N = 383^1^** | **p-value^2^** |
| --- | --- | --- | --- |
| COPD or Asthma |  |  | 0.502 |
| Absent | 62 (14%) | 368 (86%) |  |
| Present | 4 (21%) | 15 (79%) |  |
| Hypertension |  |  | 0.232 |
| Absent | 56 (14%) | 344 (86%) |  |
| present | 10 (20%) | 39 (80%) |  |
| Diabetes mellitus |  |  | 0.105 |
| absent | 55 (14%) | 345 (86%) |  |
| present | 11 (22%) | 38 (78%) |  |
| Heart disease |  |  | 0.548 |
| absent | 62 (15%) | 365 (85%) |  |
| present | 4 (18%) | 18 (82%) |  |
| Other comorbidities |  |  | 0.336 |
| absent | 51 (14%) | 315 (86%) |  |
| present | 15 (18%) | 68 (82%) |  |
| Any comorbidities |  |  | 0.039 |
| absent | 41 (13%) | 285 (87%) |  |
| present | 25 (20%) | 98 (80%) |  |
| ^1^n (%) | | | |
| ^2^Fisher's exact test; Pearson's Chi-squared test | | | |

**Table C.** Comparing the clinical manifestation between severe and non-severe dengue cases.

| **Characteristic** | **Severe**, N = 66^1^ | | **Non-severe**, N = 383^1^ | **p-value**^2^ | |
| --- | --- | --- | --- | --- | --- |
| Fever |  | |  | 0.471 | |
| absent | 1 (25%) | | 3 (75%) |  | |
| present | 65 (15%) | | 380 (85%) |  | |
| Body ache |  | |  | 0.225 | |
| absent | 30 (13%) | | 205 (87%) |  | |
| present | 36 (17%) | | 178 (83%) |  | |
| Headache |  | |  | 0.046 | |
| absent | 29 (12%) | | 219 (88%) |  | |
| present | 37 (18%) | | 164 (82%) |  | |
| Anorexia |  | |  | 0.262 | |
| absent | 38 (13%) | | 248 (87%) |  | |
| present | 28 (17%) | | 135 (83%) |  | |
| Abdominal pain |  | |  | <0.001 | |
| absent | 35 (11%) | | 293 (89%) |  | |
| present | 31 (26%) | | 90 (74%) |  | |
| Diarrhoea |  | |  | 0.151 | |
| absent | 46 (13%) | | 298 (87%) |  | |
| present | 20 (19%) | | 85 (81%) |  | |
| Cough |  | |  | 0.151 | |
| absent | 46 (13%) | | 298 (87%) |  | |
| present | 20 (19%) | | 85 (81%) |  | |
| Malaise |  | |  | 0.882 | |
| absent | 59 (15%) | | 340 (85%) |  | |
| present | 7 (14%) | | 43 (86%) |  | |
| Black stool |  | |  | 0.318 | |
| absent | 62 (14%) | | 369 (86%) |  | |
| present | 4 (22%) | | 14 (78%) |  | |
| Rash |  | |  | 0.412 | |
| absent | 62 (15%) | | 348 (85%) |  | |
| present | 4 (10%) | | 35 (90%) |  | |
| Backpain |  | |  | 0.372 | |
| absent | 52 (14%) | | 319 (86%) |  | |
| present | 14 (18%) | | 64 (82%) |  | |
| Joint pain |  | |  | 0.026 | |
| absent | 43 (13%) | | 298 (87%) |  | |
| present | 23 (21%) | | 85 (79%) |  | |
| Dehydration |  | |  | 0.492 | |
| absent | 58 (14%) | | 347 (86%) |  | |
| present | 8 (18%) | | 36 (82%) |  | |
| Respiratory distress |  | |  | 0.013 | |
| absent | 51 (13%) | | 339 (87%) |  | |
| present | 15 (25%) | | 44 (75%) |  | |
| Slurry speech |  | |  | 0.471 | |
| absent | 63 (15%) | | 371 (85%) |  | |
| present | 3 (20%) | | 12 (80%) |  | |
| Itchiness |  | |  | <0.001 | |
| absent | 53 (13%) | | 356 (87%) |  | |
| present | 13 (33%) | | 27 (68%) |  | |
| Psychiatric problem |  | |  | 0.599 | |
| absent | 66 (15%) | | 377 (85%) |  | |
| present | 0 (0%) | | 6 (100%) |  | |
| Gum bleeding |  | |  | 0.054 | |
| absent | 61 (14%) | | 373 (86%) |  | |
| present | 5 (33%) | | 10 (67%) |  | |
| Chest pain |  | |  | 0.162 | |
| absent | 55 (14%) | | 342 (86%) |  | |
| present | 11 (21%) | | 41 (79%) |  | |
| Edema |  | |  | 0.471 | |
| absent | 65 (15%) | | 380 (85%) |  | |
| present | 1 (25%) | | 3 (75%) |  | |
| vomiting |  | |  | 0.339 | |
| absent | 27 (13%) | 181 (87%) | |  |  |
| present | 39 (16%) | 202 (84%) | |  |  |
| ^1^n (%) | | | | | |
| ^2^Fisher's exact test; Pearson's Chi-squared test | | | | | |

1. ***Clinical factors measured at admission that affect dengue severity***

We used a generalized linear model with a binomial link (logistic regression) to assess the association between clinical and laboratory parameters measured at hospital admission and dengue severity. Clinical measurements at admission were obtained by linking the demographic and clinical datasets using the unique patient identifier. For each patient, the hospital admission date was extracted from the demographic dataset and merged with the clinical dataset, which contained serial measurements of systolic blood pressure (SBP), diastolic blood pressure (DBP), pulse rate, body temperature, platelet count, hematocrit, and white blood cell (WBC) count. For each clinical record, we calculated the interval between the measurement date and the admission date, defining “day 0” as the date of admission, “day −1” as one day before admission, and “day +1” as one day after admission. Only measurements taken on day 0 were retained and used as “on‑admission” values in the analyses; observations outside this window were excluded. The model was adjusted for demographic and clinical covariates, including age, body mass index (BMI), hematocrit, vital signs, and other relevant clinical variables. In this model, only log‑transformed platelet count was significantly associated with dengue severity (Table S4). The odds ratio for log‑transformed platelet count was 0.437 (95% CI 0.242–0.790), indicating that higher platelet counts at admission were associated with lower odds of severe dengue; specifically, each one‑unit increase in log platelet count was associated with an estimated 56% reduction in the odds of severe dengue after adjustment.

**Table D.** Factors affecting dengue severity. For clinical factors measured at the time of admission, we conducted an additional analysis using a logistic regression model. Odds ratios (OR), 95% confidence intervals (CI), and p-values are reported for both univariate (left) and multivariate (right) models. Platelet count and white blood cell (WBC) count were log-transformed in the model.

| Factors | Unadjusted OR (95% CI) | p-value | Adjusted OR (95% CI) | p-value |
| --- | --- | --- | --- | --- |
| Age (year) | 1.0179 (1.0021, 1.0339) | 0.0266 | 1.0062 (0.9772, 1.0360) | 0.6810 |
| BMI (kg/m^2^) | 1.0479 (0.9773, 1.1235) | 0.1885 | 1.0215 (0.9415, 1.1083) | 0.6095 |
| SBP (mmHg) | 1.0249 (1.0009, 1.0495) | 0.0417 | 1.0004 (0.9485, 1.0551) | 0.9885 |
| DBP (mmHg) | 1.0403 (1.0034, 1.0786) | 0.0321 | 1.0390 (0.9633, 1.1206) | 0.3217 |
| Pulse rate (bpm) | 0.9887 (0.9586, 1.0198) | 0.4723 | 0.9902 (0.9356, 1.0479) | 0.7328 |
| Body temperature (°F) | 0.9892 (0.4955, 1.9747) | 0.9754 | 1.1810 (0.4170, 3.3444) | 0.7541 |
| Platelet ($\mu L^{-1}$) | 0.4874 (0.3273, 0.7259) | 0.0004 | 0.4371 (0.2418, 0.7901) | 0.0061 |
| Hematocrit (%) | 0.1258 (0.0003, 1.5030) | 0.4977 | 0.0017 (0.0000, 15.127) | 0.1690 |
| WBC ($\mu L^{-1}$) | 1.6569 (0.8265, 3.3218) | 0.1548 | 1.0817 (0.4109, 2.8480) | 0.8736 |

2. ***Association between clinical factors measured at admission and blood transfusion requirements.***

Based on the logistic regression analysis of clinical factors measured at admission (Table S5), several variables were significantly associated with blood transfusion requirements in dengue cases. In univariate models, increasing age (OR = 1.0154, 95% CI = 1.0008–1.0302, p = 0.0387), SBP (OR = 1.0369, 95% CI = 1.0143–1.0600, p = 0.0013), DBP (OR = 1.0603, 95% CI = 1.0248–1.0971, p = 0.0008), lower platelet counts (OR = 0.2201, 95% CI = 0.1399–0.3463, p < 0.0001), and higher WBC counts (OR = 4.5471, 95% CI = 2.2965–9.0031, p < 0.0001) were significantly associated with transfusion requirement. However, in multivariate models, only platelet count remained a strong independent predictor (adjusted OR = 0.1235, 95% CI = 0.0551–0.2771, p < 0.0001), indicating that lower platelet counts at admission substantially increased the odds of requiring transfusion. Other factors, including age, SBP, DBP, and WBC count, lost statistical significance after adjustment, suggesting that their effects may be mediated through platelet count or other correlated variables.

**Table E.** Factors affecting blood transfusion requirements of dengue cases. For clinical factors measured at the time of admission, we conducted an additional analysis using a logistic regression model. Odds ratios (OR), 95% confidence intervals (CI), and p-values are reported for both univariate (left) and multivariate (right) models. Platelet count and white blood cell (WBC) count were log-transformed in the model. BMI: body mass index, SBP: systolic blood pressure, DBP: diastolic blood pressure, WBC: white blood cell.

| Factors | Unadjusted OR (95% CI) | p-value | Adjusted OR (95% CI) | p-value |
| --- | --- | --- | --- | --- |
| Age (year) | 1.0154 (1.0008-1.0302) | 0.0387 | 1.0059 (0.9742-1.0387) | 0.7176 |
| BMI (kg/m^2^) | 1.0132 (0.9426-1.0891) | 0.7223 | 0.9664 (0.8786-1.0631) | 0.4824 |
| SBP (mmHg) | 1.0369 (1.0143-1.0600) | 0.0013 | 0.9923 (0.9359-1.0521) | 0.7964 |
| DBP (mmHg) | 1.0603 (1.0248-1.0971) | 0.0008 | 1.0411 (0.956-1.1337) | 0.3549 |
| Pulse rate (bpm) | 0.9862 (0.9584-1.0148) | 0.3407 | 1.0473 (0.9844-1.1142) | 0.1434 |
| Body temperature (°F) | 0.7997 (0.3434-1.8619) | 0.6041 | 0.8658 (0.2873-2.6094) | 0.7979 |
| Platelet ($\mu L^{-1}$) | 0.2201 (0.1399-0.3463) | 0.0000 | 0.1235 (0.0551-0.2771) | 0.0000 |
| Hematocrit (%) | 1.4046 (0.0055-3.2888) | 0.9044 | 0.6001 (0.030-13.0547) | 0.1447 |
| WBC ($\mu L^{-1}$) | 4.5471 (2.2965-9.0031) | 0.0000 | 2.463 (0.8486-7.1487) | 0.0973 |
