# Supplementary material for: Clinical and haematological correlates of severe dengue using 2023 epidemic data: A multicentre tertiary hospital-based analysis from Dhaka, Bangladesh
Source: PLoS Negl Trop Dis. 2026 Jul 30;20(7):e0013729. doi: 10.1371/journal.pntd.0013729 (PMC13440845; doi:10.1371/journal.pntd.0013729)
Supplement: Supplementary file 2 [file pntd.0013729.s002.docx]

The authors have worked on a very timely topic. However, I have a major question regarding this study: what is new in your findings? Most of the factors identified in the study are already well-known.

I have several observations:

1. From the title, it is assumed that the authors are targeting correlated factors only. However, the aim seems to be predictor identification, which creates confusion about the focus of the study.
2. In the abstract, the authors mention a relationship with lower body mass index (BMI), but in the results section, higher BMI is described. Uniformity should be maintained throughout the manuscript.
3. In the abstract results, the factors are described as associated with dengue severity, not as predictors.
4. The study methods are unclear. The targeted population is not specified — are the participants adults or children?
5. From the methods section, the initial impression is that retrospective data analysis was performed. However, the authors also collected information directly from patients, which suggests a cross-sectional study.
6. Using a cross-sectional study design, it is not possible to identify predictors; only associations or correlations can be assessed.
7. The authors define different dengue stages according to WHO criteria, but it is not mentioned how many cases were classified as severe and based on which features.
8. In the analysis, it is not clear how adjusted factors were identified.
9. In the results section, the initial paragraph highlights the country scenario, but this is not a result of the study. It should be part of the background, as the authors did not collect total country-level cases or mortality data.
10. The results indicate that a few cases were under 18 years old. How was consent obtained for these participants? Was there an age threshold for assent or parental consent? This is not described in the methods.
11. The temporal profile of clinical factors since symptom onset is discussed, but mostly biochemical and hematological factors are reported rather than clinical signs.
12. In the temporal relation results, lower hematocrit is mentioned for both groups, but in the severity assessment, higher hematocrit is reported. There is inconsistency in the results.
13. In the discussion, lower BMI is highlighted, even though the results indicate higher BMI.
14. For BMI references, the authors use data from pediatric populations, while most study participants are adults, where the scenario may differ.
15. There is redundancy of information in the conclusion.
