# Supplementary material for: Clinical and haematological correlates of severe dengue using 2023 epidemic data: A multicentre tertiary hospital-based analysis from Dhaka, Bangladesh
Source: PLoS Negl Trop Dis. 2026 Jul 30;20(7):e0013729. doi: 10.1371/journal.pntd.0013729 (PMC13440845; doi:10.1371/journal.pntd.0013729)
Supplement: Supplementary file 3 [file pntd.0013729.s004.docx]

**Point-by-point Responses:**

**Title:** Clinical and haematological correlates of severe dengue using 2023 epidemic data: a multicentre tertiary hospital-based analysis from Dhaka, Bangladesh

**MS No:** PNTD-D-25-01995R1

**Reviewers' Comments:**

***Reviewer #1***

***Methods***

***Comment 1.1.*** *The WHO 2009 severity classification is mentioned later in the Introduction and results, but it should be explicitly stated at the start of the Methods section for clarity.*

**Response**: Thank you for your comments and suggestion. Accordingly, the severity classification based on WHO 2009 is further clearly stated in the Methods section under subsection “***Defining dengue cases and other variables***” subsection. The revised text is given as below:

“Dengue cases were classified according to the WHO guidelines into dengue (with or without warning signs) and severe dengue [1]. Suspected cases were defined as patients presenting with fever (≥100.4°F) lasting 2-7 days accompanied by at least two of the following:  headache, retro-orbital pain, myalgia, arthralgia, rash, hemorrhagic manifestations, or leukopenia. Laboratory confirmation was obtained through NS1 antigen testing for samples collected within the first five days of fever onset or IgM antibody testing for samples collected after five days [2]. Severe dengue was defined by the presence of at least one of the following criteria: (a) *Severe plasma leakage*, determined by significant fluid buildup in the body, which can lead to complications such as swelling in the abdomen or lungs. This condition may be accompanied by an increased heart rate, low blood pressure and/or a narrow difference between systolic and diastolic blood pressure, suggesting critical drop in blood volume. (b) *Severe hemorrhage*, involves serious bleeding that can pose life-threatening risk. Symptoms may include vomiting blood, passing fresh blood in the stool, or having dark, tarry stools. These bleeding episodes are often associated with unstable blood pressure and a rapid drop in hemoglobin levels, further complicating the patient’s condition. (c) *Severe organ impairment*, signifies the dysfunction of vital organs and can manifest as confusion or altered consciousness, severe liver damage (indicated by significantly elevated levels of liver enzymes, ALT or AST), or inflammation of the heart muscle (myocarditis) [3]. Body mass index (BMI) is classified into three categories: underweight (<18.5), normal (18.5-24.9), and overweight (≥25). The length of hospital stay is the period from admission to discharge. Patients with previous medical conditions such as COPD, Asthma, Hypertension, Diabetes mellitus, Heart disease, or other comorbidities are considered to have comorbidities.”

***Comment 1.2.*** *The analysis includes blood transfusion requirement and hematocrit elevation as predictors of severe dengue, reporting extremely high ORs (~15-fold). However, these variables are collected during hospitalization, and may represent clinical consequences or interventions driven by severity, rather than true early predictors. This introduces high risk of reverse causation and should be carefully reframed in the discussion and conclusions.*

Response: Thank you for the constructive comment. We agree with reviewer on the reverse causality in using blood transfusion requirement as a predictor of severe dengue, given that transfusion is typically administered after clinical deterioration. Accordingly, in the revised analyses, we have removed the blood transfusion variable from the primary predictive model of dengue severity, ensuring that only factors known at the time of admission were included as predictors. Instead, we conducted a separate analysis treating blood transfusion requirement as a clinical outcome variable. The results of this analysis, including univariate and multivariate logistic regression models based on admission variables, are presented in Supplementary Table S5.

***Results***

***Comment 1.3.*** *Age OR is reported as “1.77 per year”, but earlier the abstract reported OR=1.77 without specifying the unit. The per-unit vs. categorical age modeling must be clarified. ORs are reported without confidence intervals, which is required. Please add 95% CIs for all OR estimates.*

**Response:** Thank you for pointing this out. In the revised manuscript, we have clarified the age modeling approach, specifying that age represents a continuous variable not a categorical one. We have also ensured consistency between the abstract and the main text. Furthermore, 95% confidence intervals have now been reported for all odds ratio estimates throughout the manuscript.

***Comment 1.4.*** *Some figure captions contain typographical issues such as “severe non-severe”, “ L!” formatting artifacts, and inconsistent units (Fahrenheit vs Celsius), which should be standardized and cleaned.*

**Response:** Thank you for highlighting these issues. In the revised manuscript, we have corrected all typographical errors in the figure captions, removed formatting artifacts, and standardized the presentation of units.

***Conclusion***

***Comment 1.5.*** *There is a major inconsistency in BMI findings. The Results section states overweight individuals had a higher prevalence of severe dengue (p=0.022). Yet Table 1 (mixed-effects model) lists BMI as a strongly protective factor (OR=0.11, p<0.001). The Conclusion also emphasizes lower BMI as a risk factor. These statements contradict each other. This needs reconciliation, verification against the original data, and a corrected interpretation.*

**Response:** Thank you for the comment. As shown in the revised Table 1 in the main text, BMI was entered as a continuous variable in both the univariable and multivariable mixed-effects models, with higher values indicating higher BMI. In the univariable analysis, higher BMI was positively associated with severe dengue (uOR 1.392, 95% CI 1.185–1.634, p<0.001), consistent with the descriptive finding that severe dengue was more frequent among patients with BMI ≥25 kg/m² (Supplementary Table S1). However, in the multivariable model that simultaneously adjusted for age, blood pressure, pulse rate, temperature, platelet count, hematocrit, and WBC, the association between BMI and severe dengue reversed direction (aOR 0.094, 95% CI 0.094–0.094, p<0.001), suggesting that, conditional on these other factors, higher BMI is associated with lower odds of severe disease. These multivariable analysis is in theory to assess such observed association (in univariate analysis) under other potential factors for their confounding and causality impacts in ecological setting.

***Editorial and Data Presentation Modifications?***

***Reviewer #1:*** (No Response)

***Summary and General Comments***

***Comment 1.6.*** *This manuscript presents valuable multicentre clinical data from the 2023 Dhaka dengue epidemic, a major public health crisis. The topic is highly relevant and fits the journal scope well. The dataset is substantial and the authors attempt both admission-based and longitudinal severity modeling. However, several key issues should be addressed:*

*The WHO 2009 severity classification is mentioned later in the Introduction and results, but it should be explicitly stated at the start of the Methods section for clarity.*

**Response**: The severity classification based on WHO 2009 is also given in the Methods section under “***Defining dengue cases and other variables***” subsection. Please refer to the response of the ***comment 1.2*** above.

***Comment 1.7.*** *The analysis includes blood transfusion requirement and hematocrit elevation as predictors of severe dengue, reporting extremely high ORs (~15-fold). However, these variables are collected during hospitalization, and may represent clinical consequences or interventions driven by severity, rather than true early predictors. This introduces high risk of reverse causation and should be carefully reframed in the discussion and conclusions.*

**Response**: Yes, we agree of such risk of reverse causation. In the revised excluded blood transfusion variable from the primary predictive model of dengue severity, ensuring that only factors known at the time of admission were included as predictors. We conducted a separate analysis treating blood transfusion requirement as a clinical outcome variable (Supplementary Table S5). For details, please refer to the response of the ***comment 1.2*** above.

***Comment 1.8.*** *There is a major inconsistency in BMI findings. The Results section states overweight individuals had a higher prevalence of severe dengue (p=0.022). Yet Table 1 (mixed-effects model) lists BMI as a strongly protective factor (OR=0.11, p<0.001). The Conclusion also emphasizes lower BMI as a risk factor. These statements contradict each other. This needs reconciliation, verification against the original data, and a corrected interpretation.*

**Response**: Please refer to response to the comment # ***Comment 1.5.***

***Comment 1.9.*** *Age OR is reported as “1.77 per year”, but earlier the abstract reported OR=1.77 without specifying the unit. The per-unit vs. categorical age modeling must be clarified. ORs are reported without confidence intervals, which is required. Please add 95% CIs for all OR estimates.*

**Response**: Please refer to the response to the comment # ***Comment 1.3~~.~~***

***Comment 1.10.*** *Some figure captions contain typographical issues such as “severe non-severe”, “ L!” formatting artifacts, and inconsistent units (Fahrenheit vs Celsius), which should be standardized and cleaned.*

**Response**: Please refer to the first reviewer’s comment # ***Comment 1.4.*** *~~.~~*

***Comment 1.11.*** *The manuscript contains several language issues that require revision (examples include: multi-canter, “were found to be higher prevalence were found”, grammar, tense inconsistency, and spacing issues). These should be corrected for publication quality.*

**Response**: Thank you for noting these language and formatting issues. In the revised manuscript, we have thoroughly reviewed and corrected all grammatical errors, tense inconsistencies, spacing problems, and typographical mistakes

***Comment 1.12.*** *Overall Recommendation: The manuscript is potentially acceptable after revision. The clinical insights are useful, but the paper will be significantly strengthened if the authors clearly distinguish baseline predictors vs. hospitalization markers, correct the BMI contradictions, add confidence intervals, and improve methodological transparency and language quality.*

**Response**: Thank you for your recommendations. We have revised manuscript as per your suggestions and addressing your comments. The corresponding changes have been highlighted in blue for ease of reference.

***Reviewer #2:***

***Methods***

***Comment 2.1.*** *There is a bit of a contradiction in how the study is described: it’s labeled as a ‘retrospective review,’ but the use of written consent and questionnaires suggests a more active, prospective process. It would be really helpful if the authors could clear up exactly how the study was built specifically, were patients recruited in real-time or was this a look back at a specific window? Please also clarify the timing of the interviews (for instance, were they done right at admission?) and provide a simple breakdown of which data came from the patients’ own reports versus what was pulled from their medical charts. This context is key for readers to weigh the risk of things like recall bias on symptom timing versus the objective data found in the records.*

**Response**: Thank you for this important observation, and we apologize for the lack of clarity in the original manuscript. We have revised the text for the ***Study design, setting, and participants*** section to clearly describe the study procedures, as follows:

“ This study employed a descriptive observational design [13] and was conducted across four tertiary hospitals in Dhaka City, Bangladesh. The participating hospitals included three branches of Islami Bank Hospital (located in Kakrail, Mugda, and Mirpur) and Enam Medical College Hospital in Savar. Data collection took place from September 1 to October 31, 2023, during the peak dengue epidemic in Dhaka. All dengue patients admitted to the designated wards during this period were considered eligible. Patients were included if they had laboratory-confirmed dengue infection, defined by a positive IgM antibody test and/or NS1 antigen test. Written consent was obtained from each hospital administration to access the dengue wards. Additionally, informed consent was obtained from all patients (or from parents/guardians for minors under 18 years) prior to interviews. A total of 466 laboratory-confirmed dengue patients were enrolled in the study.

In each hospital, two trained data collectors were responsible for data collection during the study period. They were present in the hospitals during daytime hours to gather data. For consenting patients, face-to-face interviews were conducted using a semi-structured questionnaire to collect demographic information (e.g., age, sex, occupation), medical history (e.g., previous health conditions, comorbidities, COVID-19 vaccination status), and clinical manifestations (e.g., symptoms at presentation and during illness). Laboratory data, including test results, disease severity, repeated hematological measurements, vital signs, and liver function tests, were extracted from medical records. Patients were followed throughout their hospitalization, with clinical and laboratory data updated and recorded until discharge.”

***Comment 2.2.*** *The Methods and Results sections seem to be speaking two different languages regarding diagnosis. While the Methods focus on NS1, the Results bring in IgM positivity. To make this clear for the reader, could you provide a precise definition for laboratory confirmation?*

**Response**: Thank you for your comments. Both the diagnoses were considered depending on the situations. This has been included in the method section as stated below.

“Suspected cases were defined as patients presenting with fever (≥100.4°F) lasting 2-7 days accompanied by at least two of the following: headache, retro-orbital pain, myalgia, arthralgia, rash, hemorrhagic manifestations, or leukopenia. Laboratory confirmation was obtained through NS1 antigen testing for samples collected within the first five days of fever onset or IgM antibody testing for samples collected after five days.”

***Results***

***Comment 2.3.*** *There is a confusing contradiction in the data regarding BMI. The descriptive results show that severe dengue is more common in patients with a BMI of 25 or higher, but the model suggests that a higher BMI is actually protective, while the written narrative points toward lower BMI being the issue. Could you please double-check the coding and reference groups to ensure everything is aligned? It would also be helpful to clarify the exact definitions used for severe vs. non-severe outcomes.*

**Response:** Thank you for this insightful comment. We have carefully re-checked the coding of BMI, the specification of the outcome, and the reference groups in all analyses and can confirm that they are correctly aligned. As shown in Table 1 in the main text, BMI was entered as a continuous variable in both the univariable and multivariable mixed-effects models, with higher values indicating higher BMI. In the univariable analysis, higher BMI was positively associated with severe dengue (OR 1.392, 95% CI 1.185–1.634, p<0.001), consistent with the descriptive finding that severe dengue was more frequent among patients with BMI ≥25 kg/m². However, in the multivariable model that simultaneously adjusted for age, blood pressure, pulse rate, temperature, platelet count, hematocrit, and WBC, the association between BMI and severe dengue reversed direction (adjusted OR 0.094, 95% CI 0.094–0.094, p<0.001), suggesting that, conditional on these other factors, higher BMI is associated with lower odds of severe disease. This apparent contradiction reflects confounding and the differing nature of crude versus adjusted associations rather than a coding error; the descriptive comparison and the multivariable OR answer related but distinct questions. Please also refer to the responses to the first reviewer’s comment # ***Comment 1.5,*** *for details.*

***Comment 2.4.*** *Could you give us a clearer look at the big picture? It would be great to see the total number and percentage of severe dengue cases, both overall and split by site. Including confidence intervals here would really help, as it provides the necessary "anchor" for your models and plots. Without those baseline numbers, it’s a little tough for readers to fully grasp the scale and context of the results you’re showing.*

**Response:** Thank you for this helpful suggestion. The total number and percentage of severe dengue cases by site are already presented in the supplementary material; please refer to Tables S1–S3 for these data. Regarding the regression results, all model-based estimates are now presented with their corresponding 95% confidence intervals and p‑values in the revised tables (Table 1). We agree with reviewer that including the confidence intervals improves interpretability and have therefore added them in the revised manuscript.

***Comment 2.5.*** *The current analysis treats blood transfusions as a predictor of severe dengue, but since a transfusion is usually a response to a patient getting clinically worse, it likely happened after the severity was already established. This creates a "reverse causality" issue where the transfusion is more of a marker of the disease's progress than a predictor of it. Could you clarify the timing of these transfusions? It might be cleaner to remove them from your predictive models or treat them separately as a clinical outcome, keeping your "predictor" variables strictly to what was known at the time of admission.*

**Response:** We acknowledge the reviewer’s concern regarding the potential for reverse causality in using blood transfusion requirement as a predictor of severe dengue, given that transfusion is typically administered after clinical deterioration. To address this, we have removed the blood transfusion variable from the primary predictive model of dengue severity, ensuring that only factors known at the time of admission were included as predictors. Instead, we conducted a separate analysis treating blood transfusion requirement as a clinical outcome variable. The results of this analysis, including univariate and multivariate logistic regression models based on admission variables, are presented in Supplementary Table S5. Please also refer to the response of the first reviewer’s comment # ***Comment 1.2.***

***Comment 2.6.*** *The logistic regression is described as using admission values, but several covariates look like they may represent events or measurements during hospitalisation period. Please clearly define the starting time for each variable used in each model.*

**Response:** We apologise for the lack of clarity on this point. All covariates in the logistic regression were defined with respect to the hospital admission date and restricted to baseline values. For consenting patients, face-to-face interviews were conducted using a semi-structured questionnaire to collect demographic information (e.g., age, sex, occupation), medical history (e.g., previous health conditions, comorbidities, COVID-19 vaccination status), and clinical manifestations (e.g., symptoms at presentation and during illness). Laboratory data, including test results, disease severity, repeated hematological measurements, vital signs, and liver function tests, were extracted from medical records. As described in the revised Methods (see details in the Reviewer 2;s ***Comment #2.1***) and Supplementary text for each clinical measurements we calculated the interval between the measurement date and the admission date, defining “day 0” as the date of admission, “day −1” as one day before admission, and “day +1” as one day after admission. Only measurements taken on day 0 (zero) were retained and used as “on‑admission” values for systolic and diastolic blood pressure, pulse rate, body temperature, platelet count, hematocrit, and white blood cell count; observations outside this window were excluded from the regression analysis. No events or measurements occurring after admission (beyond day 0) were used as predictors in the models. This clarification has now been added to the Supplementary Methods (Section 1).

***Comment 2.7.*** *Please ensure tables specify units and denominators, include missingness, and provide full model outputs including sample size per model, CIs, and p-values*

**Response:** We have revised Table 1 to address these points. Units are now specified for all variables, and the number of observations with missing data is reported. For the regression analyses, we now provide full model outputs, including the sample size for each model, odds ratios with 95% confidence intervals, and corresponding p‑values.

***Conclusion***

***Comment 2.8.*** *Conclusions about BMI and other factors must match the corrected descriptive and multivariable results after the authors reconcile inconsistencies*

**Response:** Thank you for this insightful comment. Please refer to Reviewer 1’s ***Comment #1.5*** and Reviewer 2’s ***Comment #2.3*** for details.

“We have carefully re-checked the coding of BMI, the specification of the outcome, and the reference groups in all analyses and can confirm that they are correctly aligned. As shown in Table 1 in the main text, BMI was entered as a continuous variable in both the univariable and multivariable mixed-effects models, with higher values indicating higher BMI. In the univariable analysis, higher BMI was positively associated with severe dengue (OR 1.392, 95% CI 1.185–1.634, p<0.001), consistent with the descriptive finding that severe dengue was more frequent among patients with BMI ≥25 kg/m². However, in the multivariable model that simultaneously adjusted for age, blood pressure, pulse rate, temperature, platelet count, hematocrit, and WBC, the association between BMI and severe dengue reversed direction (adjusted OR 0.094, 95% CI 0.094–0.094, p<0.001), suggesting that, conditional on these other factors, higher BMI is associated with lower odds of severe disease. This apparent contradiction reflects confounding and the differing nature of crude versus adjusted associations rather than a coding error; the descriptive comparison and the multivariable OR answer related but distinct questions.”

***Comment 2.9.*** *It would be helpful to expand the limitations section to give the results more context. Specifically, please address how focusing on tertiary-care cases might introduce selection bias, and how recall bias could affect symptom timing. It’s also important to mention any missing data in the repeated labs and how clinical management decisions (like treatment timing) might have acted as a confounder. Briefly touching on these points will really help clarify the scope and reliability of the findings.*

**Response:** We appreciate the reviewer’s suggestion and have extended the limitations section accordingly. In the revised manuscript, we now address how the focus on tertiary-care hospital cases may introduce selection bias by potentially overrepresenting more severe dengue presentations compared to cases managed in primary or community settings. Please refer to the ***Limitation*** section for details. The relevant text is provided below:

“…this study has several limitations that should be considered when interpreting the findings.

First, the study was conducted in four tertiary hospitals in Dhaka City during the peak of the 2023 dengue outbreak. As these facilities primarily manage patients with more severe illness, this focus may introduce selection bias, potentially overrepresenting severe cases compared to community or primary care settings. This could limit the generalizability of our findings to other regions, healthcare levels, or non-epidemic periods. Second, the sample size, while substantial, may have been insufficient to detect statistically significant associations for certain comorbidities and symptoms, particularly those with low prevalence. Three, the reliance on hospital records and patient interviews for data collection may introduce recall bias, especially for variables such as symptom onset and duration. Inaccurate recall could affect the precision of timing-related variables and weaken associations with outcomes. Fourth, some variables, including repeated laboratory measurements during hospitalization, had missing data due to variations in clinical practice and patient availability. This may have reduced statistical power or introduced bias if missingness was related to disease severity or treatment decisions. Fifth, clinical management decisions, such as the timing and type of treatment administered, could have acted as confounders, influencing both laboratory values and outcomes. Since treatment facilities were not same across hospitals, variations in management may have affected the associations observed. …”

***Editorial and Data Presentation Modifications?***

***Comment 2.10.*** *I recommend a minor revision*

**Response:** Thanks.

***Summary and General Comments***

***Comment 2.11.*** *This dataset from the 2023 Dhaka epidemic is really timely and could offer great clinical and public health insights, but a few inconsistencies make the findings hard to fully follow. Right now, the study design and lab criteria feel a bit vague. There are also some contradictions in the BMI data, scaling issues with the odds ratios, and no mention of how missing data was handled. Finally, using treatments like transfusions as "predictors" is a bit circular, since they usually happen after a patient has already taken a turn for the worse. Cleaning up these points would make the paper much stronger and the results far more reliable.*

**Response**: Thank you for your valuable comments. We have clarified the study design and laboratory criteria, corrected inconsistencies in BMI data, adjusted odds ratio scaling, explicitly described our approach to missing data, and revised our analysis to avoid circular inference from transfusion. Where necessary, we have added supplementary analyses and provided clear justifications for each change. Please see the Methods, Results, and Conclusion sections for our point-by-point responses.

***Reviewer #3:***

***Methods***

***Comment 3.1.*** *The study objectives are generally clear, aiming to identify demographic, clinical, and hematological correlates of severe dengue during the 2023 Dhaka outbreak.*

**Response**: Thanks.

***Comment 3.2.*** *The multicenter, hospital-based observational design is appropriate for the stated objectives. However, the study is described as both cross-sectional and as involving repeated measurements, which is conceptually inconsistent. It might be more accurate if described as a retrospective observational cohort with longitudinal follow-up during hospitalization.*

**Response**: Thank you for your suggestions. Please refer to Reviewer 2’s ***Comment #2.1*** that we have revised the ***Study design, setting, and participants*** section to clearly describe the study procedures, as follows:

“ This study employed a descriptive observational design [13] and was conducted across four tertiary hospitals in Dhaka City, Bangladesh. The participating hospitals included three branches of Islami Bank Hospital (located in Kakrail, Mugda, and Mirpur) and Enam Medical College Hospital in Savar. Data collection took place from September 1 to October 31, 2023, during the peak dengue epidemic in Dhaka. All dengue patients admitted to the designated wards during this period were considered eligible. Patients were included if they had laboratory-confirmed dengue infection, defined by a positive IgM antibody test and/or NS1 antigen test. Written consent was obtained from each hospital administration to access the dengue wards. Additionally, informed consent was obtained from all patients (or from parents/guardians for minors under 18 years) prior to interviews. A total of 466 laboratory-confirmed dengue patients were enrolled in the study.

In each hospital, two trained data collectors were responsible for data collection during the study period. They were present in the hospitals during daytime hours to gather data. For consenting patients, face-to-face interviews were conducted using a semi-structured questionnaire to collect demographic information (e.g., age, sex, occupation), medical history (e.g., previous health conditions, comorbidities, COVID-19 vaccination status), and clinical manifestations (e.g., symptoms at presentation and during illness). Laboratory data, including test results, disease severity, repeated hematological measurements, vital signs, and liver function tests, were extracted from medical records. Patients were followed throughout their hospitalization, with clinical and laboratory data updated and recorded until discharge.”

***Comment 3.3.*** *The study population is clearly described, including inclusion criteria (laboratory-confirmed dengue via NS1 or IgM positivity), setting, time frame, and hospital locations.*

**Response**: Thank you for your positive feedback.

***Comment 3.4.*** *The final analytical sample is reasonably large for a hospital-based dengue study and appears sufficient for most of the primary analyses conducted.*

**Response**: Thank you for this encouraging comment.

***Comment 3.5.*** *Overall, the statistical methods are appropriate and well-chosen. The use of chi-squared/Fisher’s exact tests for categorical variables and parametric/non-parametric tests for continuous variables is standard. The application of generalized linear mixed-effects models to repeated measures appropriately accounts for within-patient correlation and inter-hospital variability, which is a notable strength. However, the rationale for including a requirement for blood or platelet transfusion as an adjustment variable is questionable, as this may be a consequence rather than a predictor of severity. One more thing, the handling of missing data is not described, and if present, it should be clarified.*

**Response:** We acknowledge the reviewer’s concern regarding the potential for reverse causality in using blood transfusion requirement as a predictor of severe dengue, given that transfusion is typically administered after clinical deterioration. Therefore, , we have excluded blood transfusion variable from the primary predictive model of dengue severity, ensuring that only factors known at the time of admission were included as predictors. However, we conducted a separate analysis treating blood transfusion requirement as a clinical outcome variable. The results of this analysis, including univariate and multivariate logistic regression models based on admission variables, are presented in Supplementary Table S5. Please also refer to the response to the comments ***Comment #1.2 (****Reviewer 1****)*** *and* ***Comment #2.5 (****Reviewer 2****).*** Additionally, we addressed the missing data handling in the revised text as below. Details are given in the ***Statistical analysis*** subsections in the **Methods**.

“…All models were adjusted for demographic characteristics including age and BMI. Missing data were excluded from the analysis.”

***Comment 3.6.*** *The study reports ethical approval from the institutional ethical approval committee of Primeasia University, yet none of the listed authors are affiliated with Primeasia University. This raises an important ethical and procedural concern. The authors must clarify why Primeasia University served as the ethical review body; whether any author held an adjunct, visiting, or prior affiliation at Primeasia at the time of approval; and whether formal agreements existed between Primeasia University and the participating hospitals or investigators. Without clarification, this raises questions regarding ethical oversight appropriateness and institutional responsibility.*

**Response:** Thank you for raising this important point. At the time of obtaining ethical approval, Farzana Sultana Bari was a former faculty member of Primeasia University. We have clarified this in the revised manuscript and noted that the approval was granted under her prior affiliation. Please see ***Ethical declaration*** section for details.

“The Institutional Ethical Approval Committee of Primeasia University, Bangladesh (PAU/IEAC/23/123) reviewed and approved the study. Prior to conducting the interviews, written consent was obtained from each patient. At the time of obtaining ethical approval, Farzana Sultana Bari held a prior faculty affiliation with Primeasia University, which facilitated the ethical review process.”

***Results***

***Comment 3.7.*** *Overall, the analyses presented in the results section largely align with the analysis plan described in the methods. However, result section occasionally implies predictive inference, whereas much of the analysis is, I think, associative. No big issue though.*

**Response:** Thank you for this valuable observation. We have revised the wording in the Results and Discussion sections to ensure that interpretations are framed as associations rather than predictive inferences where appropriate.

***Comment 3.8.*** *This section is data-rich and generally clearly presented*.

**Response:** Thank you for your encouraging feedback.

***Comment 3.9.*** *In general, the tables and figures are of good technical quality and enhance clarity.*

**Response:** Thank you for your positive comment.

***Conclusion***

***Comment 3.10.*** *The data support the conclusions. However, variables such as blood or platelet transfusion requirement have been discussed as predictors, despite being more plausibly consequences of severe disease, which weakened causal inference. The conclusion/discussion would be more robust if they more clearly distinguished associations from predictive or causal claims.*

**Response:** We acknowledge the reviewer’s concern regarding the potential for reverse causality in using blood transfusion requirement as a predictor of severe dengue, given that transfusion is typically administered after clinical deterioration. To address this, we have removed the blood transfusion variable from the primary predictive model of dengue severity, ensuring that only factors known at the time of admission were included as predictors. Instead, we conducted a separate analysis treating blood transfusion requirement as a clinical outcome variable. The results of this analysis, including univariate and multivariate logistic regression models based on admission variables, are presented in ***Supplementar*y** Table S5. Please also refer to response to the Reviewer 1’s ***Comment #1.2,*** *Reviewer 2’s* ***Comment #2.5, and*** *Reviewer 3’s* ***Comment #3.5.***

***Comment 3.11.*** *Are the limitations of analysis clearly described? – Yes*

**Response:** Thanks.

***Comment 3.12.*** *Do the authors discuss how these data can be helpful to advance our understanding of the topic under study? – Yes*

**Response:** Thanks.

***Comment 3.13.*** *Public health relevance is clearly acknowledged and appropriately emphasized.*

**Response:** Thanks.

***Editorial and Data Presentation Modifications?***

***Comment 3.14.*** *One minor issue in discrepancy in reported national dengue burden- in the inconsistent reporting of the total number of dengue cases during the 2023 outbreak. The manuscript variously reports figures such as over 321,000 and over 321179 cases across the abstract, introduction, and results sections.*

**Response**: Thank you for your feedback. We have addressed this issue and incorporated the necessary corrections in the revised version of the manuscript.

***Summary and General Comments***

***Comment 3.15.*** *This manuscript presents a timely and policy-relevant analysis of clinical and haematological correlates of severe dengue during the 2023 epidemic in Dhaka, Bangladesh. But one thing, like 2019, 2023 is a year of DENV serotype swap (from DENV-3 to DENV-2) that was one of the reasons of so much mortality and morbidity. So, author should mention this serotype swap issue or resurgence of DENV-2 in 2023 in their manuscript with proper citation.*

**Response**: Thank you for your valuable suggestion. We have now included a description of the serotype swap from DENV-3 in 2019 to DENV-2 in 2023 in the ***Discussion*** section, highlighting its potential role in the increased morbidity and mortality observed during the outbreak. Appropriate citations have been added to support this information.

***Comment 3.16.*** *Key strengths of the study include the multicenter design, use of standardized WHO 2009 severity criteria, rich supplementary analyses.*

**Response**: Thank you for the positive feedback.

***Comment 3.17.*** *Weaknesses: Inconsistencies in epidemiological framing, particularly the varying reporting of national case counts (≈321,000); conceptual ambiguity in study design, described as cross-sectional despite the use of longitudinal repeated-measures analyses.*

**Response**: Thank you for pointing out these issues. We have corrected the national case count to 321,179 and revised the description of the study design in accordance with your suggestions.

***Comment 3.18.*** *The study offers moderate novelty. Many identified risk factors are well established in a myriad of dengue literature. The primary contribution lies in context-specific epidemiological insight from the 2023 Dhaka-based outbreak.*

**Response**: Thank you for your observation. While we acknowledge that several identified risk factors have been documented in previous dengue literature, our study offers valuable, context-specific epidemiological insights into the unprecedented 2023 outbreak in Dhaka, which occurred during a serotype shift. Our findings highlight the complex interplay of multiple factors influencing dengue severity and underscore the importance of tailored public health strategies. By identifying these risk factors within the local context, healthcare providers can improve the management of severe dengue cases and mitigate the impact of future outbreaks.

***Comment 3.19.*** *The manuscript is well written, well structured, and supported by appropriate citations. Figures and tables are of good quality and mostly clear.*

**Response**: Thank you for the encouraging feedback

***Comment 3.20.*** *No evidence of dual publication or plagiarism is apparent. However, the ethical approval obtained from Primeasia University, despite the absence of author affiliation, requires clarification. This issue raises questions regarding institutional oversight and must be explicitly addressed to meet ethical transparency standards.*

**Response**: Thank you for raising this important point. At the time of obtaining ethical approval, Farzana Sultana Bari was a former faculty member of Primeasia University. We have clarified this in the revised manuscript and noted that the approval was granted under her prior affiliation.

***Reviewer #4:***

***Methods***

***Comment 4.1.*** *Methods are not clear for the study*

**Response:** Thank you for your feedback. We apologize for the lack of clarity in the original methods section. As per the specific suggestions, we have now revised the test by providing more detailed and transparent description for the sections including the study design, procedures, and analysis. Please refer to the ***Methods*** *section* and responses to the comments bellow.

***Results***

***Comment 4.2.*** *Results are not clearly presented, and result has no new findings*

**Response:** Thank you for your comments. We have revised the results section to improve clarity and presentation. In the updated manuscript, we have refined the model outputs and clarified the protective effect of BMI in the mixed-effects model, as shown in ***Table 1*** of the main text. Additionally, we have excluded blood transfusion as a predictor of severe dengue and instead considered it as a consequence and outcome of the disease. Correspondingly, we have included additional analyses in **Supplementary Table S5** to support this change. Please refer to the responses to your specific comments below.

***Conclusions*** ***Comment 4.3.*** *yes, but not clear*

**Response:** We have updated the conclusion. Please refer to the revised manuscript.

***Editorial and Data Presentation Modifications?***

***Reviewer #4:*** (No Response)

***Summary and General Comments***

***Reviewer #4:*** (No Response)

***Reviewer 4’s comment From the attachment***

***Comment 4.4.*** *The authors have worked on a very timely topic. However, I have a major question regarding this study: what is new in your findings? Most of the factors identified in the study are already well-known.*

**Response**: Thank you for your observation. While we acknowledge that several identified risk factors have been documented in previous dengue literature, our study offers valuable, context-specific epidemiological insights into the unprecedented 2023 outbreak in Dhaka, which occurred during a serotype shift. Our findings highlight the complex interplay of multiple factors influencing dengue severity and underscore the importance of tailored public health strategies. By identifying these risk factors within the local context, healthcare providers can improve the management of severe dengue cases and mitigate the impact of future outbreaks.

***Comment 4.5.*** *I have several observations:*

*From the title, it is assumed that the authors are targeting correlated factors only. However, the aim seems to be predictor identification, which creates confusion about the focus of the study.*

**Response**: We apologize for the confusion caused by the wording. In the revised version, we have clarified that our study explores associated factors rather than solely identifying predictors and have adjusted the text accordingly.

***Comment 4.6.*** *In the abstract, the authors mention a relationship with lower body mass index (BMI), but in the results section, higher BMI is described. Uniformity should be maintained throughout the manuscript.*

**Response**: Thank you for pointing out this inconsistency. We have corrected the descriptions of BMI in both the abstract and the results section to ensure accuracy and uniformity throughout the manuscript.

***Comment 4.7.*** *In the abstract results, the factors are described as associated with dengue severity, not as predictors.*

**Response**: We apologize for the confusion caused by the wording. In the revised version, we have clarified that our study explores associated factors rather than solely identifying predictors and have adjusted the text accordingly.

***Comment 4.8.*** *The study methods are unclear. The targeted population is not specified — are the participants adults or children?*

**Response**: We have revised the Methods section to clearly specify the targeted population. All dengue patients admitted to the designated wards during the study period were considered for inclusion, as described in the “***Study Design, Setting, and Participants***” subsection. Detailed age stratification of participants is also provided in ***Supplementary Table S1***.

***Comment 4.9.*** *From the methods section, the initial impression is that retrospective data analysis was performed. However, the authors also collected information directly from patients, which suggests a cross-sectional study.*

**Response**: Thank you for your valuable observation. During the study period, two trained data collectors were present at each participating hospital. They identified currently hospitalized dengue patients from medical records. Eligibility required laboratory-confirmed dengue infection, defined by a positive IgM antibody test and/or NS1 antigen test. For consenting patients, we conducted face-to-face interviews using a semi-structured questionnaire to gather:

• Demographic information (age, sex, occupation, etc.)

• Medical history (previous health conditions, comorbidities, COVID-19 vaccination status, etc.)

• Clinical manifestations (symptoms at presentation and during illness)

Laboratory data, including test results, disease severity, repeated hematological measurements, vital signs, and liver function tests, were extracted from medical records. Patients were followed throughout their hospitalization, with clinical and laboratory data updated and recorded until discharge.

Based on this design, we classified the study as a descriptive observational study [4], and this has been reflected in the ***Methods*** section of the manuscript.

***Comment 4.10*** *Using a cross-sectional study design, it is not possible to identify predictors; only associations or correlations can be assessed.*

**Response:** Thank you for this valuable comment. We agree that with a cross-sectional study design it is not appropriate to refer to “predictors,” as causal or temporal relationships cannot be established and only associations can be assessed. In response, we have revised the wording throughout the manuscript. Specifically:

- We have replaced the term “predictors” with “risk factors” or “factors associated with dengue severity,” as appropriate.
- We now explicitly state that our analyses identify associations with dengue severity rather than predicting severity.

***Comment 4.11.*** *The authors define different dengue stages according to WHO criteria, but it is not mentioned how many cases were classified as severe and based on which features.*

**Response:** In our study, after restricting to NS1- or IgM-positive cases, 449 patients were retained for analysis. Among these, 383/449 (85.3%) were classified as non-severe dengue and 66/449 (14.7%) as severe dengue. Within the severe dengue group, 57/449 (12.7%) met criteria for dengue hemorrhagic fever and 9/449 (2.0%) had severe organ impairment.

We have clarified this in ***Results*** *section* as follows:

“After cleaning the data to confirm NS1 or IgM positivity, 449 patients were retained for the main analysis. Among these, 66/449 (14.7%) met criteria for severe dengue and 383/449 (85.3%) for non-severe dengue. Dengue hemorrhagic fever was identified in 57/449 (12.7%) patients, and severe organ impairment in 9/449 (2.0%).”

***Comment 4.12.*** *In the analysis, it is not clear how adjusted factors were identified.*

**Response**: Our primary aim was to identify associations between clinical factors and dengue severity. Therefore, we included all relevant clinical variables, along with age and BMI, in the analysis. We performed both univariate and multivariate analyses to explore these associations, using a binomial distribution under the assumption that severity followed this distribution. For repeated measures of clinical factors, we developed generalized linear mixed-effects models to account for variations within individual patients as well as between hospitals. In addition, we conducted logistic regression analyses for the same factors measured at the time of hospital admission. A separate analysis was performed to identify factors associated with the requirement for blood transfusion, which was considered an indicator of severity. Please refer to ***Table 1*** in the main text and ***Tables S4-S5*** in the ***Supplementary*** ***text***.

***Comment 4.13.*** *In the results section, the initial paragraph highlights the country scenario, but this is not a result of the study. It should be part of the background, as the authors did not collect total country-level cases or mortality data.*

**Response**: We included this description to provide context for the data collection period and to illustrate how the epidemic evolved during that time. Additionally, the paragraph highlights that Dhaka City experienced the highest mortality and morbidity during the 2023 outbreak, underscoring the importance of studying the dengue epidemic in this location. However, we acknowledge that this information is more appropriate for the ***Introduction*** section, and we have moved it there accordingly.

***Comment 4.14.*** *The results indicate that a few cases were under 18 years old. How was consent obtained for these participants? Was there an age threshold for assent or parental consent? This is not described in the methods.*

**Response**: For participants under 18 years of age, informed consent was obtained from their parents or guardians. We have revised the ***Methods*** section to clarify that informed consent was secured from all participating patients, and for minors under 18 years, consent was obtained from a parent or guardian prior to the interviews.

***Comment 4.15.*** *The temporal profile of clinical factors since symptom onset is discussed, but mostly biochemical and hematological factors are reported rather than clinical signs.*

**Response:** As stated in the ***Methods*** section, “Laboratory data, including test results, severity levels, repeated hematological measurements, vital signs, and liver function tests, were extracted from medical records.” Unfortunately, detailed clinical signs were not consistently documented in a temporal format within the medical records. Therefore, it was not possible to collect and analyze clinical signs over time in the same manner as biochemical and hematological parameters.

***Comment 4.16.*** *In the temporal relation results, lower hematocrit is mentioned for both groups, but in the severity assessment, higher hematocrit is reported. There is inconsistency in the results.*

**Response:** In the temporal analysis, we compared hematocrit levels between dengue groups over time. Although hematocrit declined in both groups, the differences between them remained statistically significant across the time points, with the severe group consistently showing lower hematocrit than the non-severe group. In the severity assessment (***Table 1***), the revised generalized mixed-effects model, adjusted for relevant covariates, indicated that higher hematocrit was associated with lower odds of severe disease. The difference in findings arises because the temporal analysis examines changes and differences over time, whereas the severity assessment evaluates the association between hematocrit and severity status in an adjusted model.

***Comment 4.17.*** *In the discussion, lower BMI is highlighted, even though the results indicate higher BMI.*

**Response:** The statement in the discussion refers to the findings from the multivariable regression model, where lower BMI was associated with severity after adjusting for other factors. In contrast, the unadjusted results from the univariate analysis indicated that higher BMI was associated with severe dengue. The difference arises because the regression model accounts for potential confounding variables, which can alter the direction or magnitude of the association observed in the univariate analysis.

***Comment 4.18.*** *For BMI references, the authors use data from pediatric populations, while most study participants are adults, where the scenario may differ.*

**Response:** We acknowledge the reviewer’s observation regarding the predominance of adult participants in our study. In the revised manuscript, we have included an additional reference reporting BMI association with dengue severity in adult populations to ensure the discussion reflects evidence relevant to our cohort. The statement is given as below:

“For example, a recent systematic review reported that patient with obesity had a 50% higher likelihood of developing severe dengue (OR = 1.50, 95% CI: 1.15–1.97) [21].”

***Comment 4.19.*** *There is redundancy of information in the conclusion.*

**Response:** We have revised the conclusion as below,

“This study identified key demographic and clinical factors associated with severe dengue in the 2023 Dhaka epidemic. Older age was consistently and strongly linked to severe disease, even after adjusting for other predictors. In the multivariable model, lower BMI emerged as a significant risk factor, suggesting that undernutrition or altered metabolic status may contribute to poorer outcomes. Higher DBP and pulse rate were independently associated with increased odds of severe dengue, while higher SBP, body temperature, platelet count, hematocrit, and WBC count were associated with lower odds, indicating potential protective or compensatory physiological responses. Delays in hospital admission, prolonged hospitalization, and extended recovery times were also strongly linked to severe outcomes, emphasizing the importance of timely medical intervention. The presence of comorbidities and specific symptoms, such as abdominal pain, respiratory distress, and itchiness, further underscored the complexity of dengue progression.”

**References**

1.	World Health Organization. Dengue: guidelines for diagnosis, treatment, prevention and control: Department of Control of Neglected Tropical Diseases, WHO; 2009.

2.	Directorate General of Health Services. National Guideline for Clinical Management of Dengue Syndrome. In: National Malaria Elimination and Aedes Transmitted Diseases Control Program Disease Control Unit, editor. 4 ed2018.

3.	Kuo HJ, Lee IK, Liu JW. Analyses of clinical and laboratory characteristics of dengue adults at their hospital presentations based on the World Health Organization clinical-phase framework: Emphasizing risk of severe dengue in the elderly. J Microbiol Immunol Infect. 2018;51(6):740-8. Epub 20170622. doi: 10.1016/j.jmii.2016.08.024. PubMed PMID: 28734676.

4.	Grimes DA, Schulz KF. An overview of clinical research: the lay of the land. The Lancet. 2002;359(9300):57-61. doi: 10.1016/S0140-6736(02)07283-5.
