# Supplementary material for: Clinical and haematological correlates of severe dengue using 2023 epidemic data: A multicentre tertiary hospital-based analysis from Dhaka, Bangladesh
Source: PLoS Negl Trop Dis. 2026 Jul 30;20(7):e0013729. doi: 10.1371/journal.pntd.0013729 (PMC13440845; doi:10.1371/journal.pntd.0013729)
Supplement: Peer review history

# Peer review: Decision Letter 0

**DOI:** 10.1371/journal.pntd.0013729.r001

**Authors:** Álvaro Acosta-Serrano, Md. Kamrujjaman

2 Mar 2026

-->PNTD-D-25-01995

Clinical and haematological correlates of severe dengue using 2023 epidemic data: a multicentre tertiary hospital-based analysis from Dhaka, Bangladesh

PLOS Neglected Tropical Diseases

Dear Dr. Ali,

Thank you for submitting your manuscript to PLOS Neglected Tropical Diseases. After careful consideration, we feel that it has merit but does not fully meet PLOS Neglected Tropical Diseases's publication criteria as it currently stands. Therefore, we invite you to submit a revised version of the manuscript that addresses the points raised during the review process.

Please submit your revised manuscript within by May 01 2026 11:59PM. If you will need more time than this to complete your revisions, please reply to this message or contact the journal office at plosntds@plos.org. When you're ready to submit your revision, log on to https://www.editorialmanager.com/pntd/ and select the 'Submissions Needing Revision' folder to locate your manuscript file.

Please include the following items when submitting your revised manuscript:

* A letter that responds to each point raised by the editor and reviewer(s). You should upload this letter as a separate file labeled 'Response to Reviewers'. This file does not need to include responses to any formatting updates and technical items listed in the 'Journal Requirements' section below.

* A marked-up copy of your manuscript that highlights changes made to the original version. You should upload this as a separate file labeled 'Revised Manuscript with Track Changes'.

* An unmarked version of your revised paper without tracked changes. You should upload this as a separate file labeled 'Manuscript'.

If you would like to make changes to your financial disclosure, competing interests statement, or data availability statement, please make these updates within the submission form at the time of resubmission. Guidelines for resubmitting your figure files are available below the reviewer comments at the end of this letter.

We look forward to receiving your revised manuscript.

Kind regards,

Md. Kamrujjaman

Academic Editor

PLOS Neglected Tropical Diseases

Álvaro Acosta-Serrano

Section Editor

PLOS Neglected Tropical Diseases

Shaden Kamhawi

co-Editor-in-Chief

PLOS Neglected Tropical Diseases

orcid.org/0000-0003-4304-636XX

Paul Brindley

co-Editor-in-Chief

PLOS Neglected Tropical Diseases

orcid.org/0000-0003-1765-0002

**Journal Requirements:**

If the reviewer comments include a recommendation to cite specific previously published works, please review and evaluate these publications to determine whether they are relevant and should be cited. There is no requirement to cite these works unless the editor has indicated otherwise.

1) Please ensure that the CRediT author contributions listed for every co-author are completed accurately and in full.

At this stage, the following Authors/Authors require contributions: M. Pear Hossain, Rudbar Mahmood, Farzana Sultana Bari, Afrin Naher Kana, Bibek Ahamed, Munia Afroza Shanta, Sadia Afrin, Musleha Akter, Tahmina Akter Anika, Mehejabin Nurunnahar, Sayeda Jahin Tasnim, Abu Bakkar Siddique, Linyan Li, and Sheikh Taslim Ali. Please ensure that the full contributions of each author are acknowledged in the "Add/Edit/Remove Authors" section of our submission form.

The list of CRediT author contributions may be found here: https://journals.plos.org/plosntds/s/authorship#loc-author-contributions

2) We ask that a manuscript source file is provided at Revision. Please upload your manuscript file as a .doc, .docx, .rtf or .tex. If you are providing a .tex file, please upload it under the item type u2018LaTeX Source Fileu2019 and leave your .pdf version as the item type u2018Manuscriptu2019.

3) Please upload all main figures as separate Figure files in .tif or .eps format. For more information about how to convert and format your figure files please see our guidelines:

https://journals.plos.org/plosntds/s/figures

4) Some material included in your submission may be copyrighted. According to PLOSu2019s copyright policy, authors who use figures or other material (e.g., graphics, clipart, maps) from another author or copyright holder must demonstrate or obtain permission to publish this material under the Creative Commons Attribution 4.0 International (CC BY 4.0) License used by PLOS journals. Please closely review the details of PLOSu2019s copyright requirements here: PLOS Licenses and Copyright. If you need to request permissions from a copyright holder, you may use PLOS's Copyright Content Permission form.

Please respond directly to this email and provide any known details concerning your material's license terms and permissions required for reuse, even if you have not yet obtained copyright permissions or are unsure of your material's copyright compatibility. Once you have responded and addressed all other outstanding technical requirements, you may resubmit your manuscript within Editorial Manager.

Potential Copyright Issues:

i) Figures 1C, 1D, and 1E. Please (a) provide a direct link to the base layer of the map (i.e., the country or region border shape) and ensure this is also included in the figure legend; and (b) provide a link to the terms of use / license information for the base layer image or shapefile. We cannot publish proprietary or copyrighted maps (e.g. Google Maps, Mapquest) and the terms of use for your map base layer must be compatible with our CC BY 4.0 license.

Note: if you created the map in a software program like R or ArcGIS, please locate and indicate the source of the basemap shapefile onto which data has been plotted.

If your map was obtained from a copyrighted source please amend the figure so that the base map used is from an openly available source. Alternatively, please provide explicit written permission from the copyright holder granting you the right to publish the material under our CC BY 4.0 license.

If you are unsure whether you can use a map or not, please do reach out and we will be able to help you. The following websites are good examples of where you can source open access or public domain maps:

* U.S. Geological Survey (USGS) - All maps are in the public domain. (http://www.usgs.gov)

* PlaniGlobe - All maps are published under a Creative Commons license so please cite u201cPlaniGlobe, http://www.planiglobe.com, CC BY 2.0u201d in the image credit after the caption. (http://www.planiglobe.com/?lang=enl)

* Natural Earth - All maps are public domain. (http://www.naturalearthdata.com/about/terms-of-use/).

5) In the online submission form, you indicated that Data used in this manuscript can be obtained from the corresponding author upon reasonable request.. All PLOS journals now require all data underlying the findings described in their manuscript to be freely available to other researchers, either

1. In a public repository

2. Within the manuscript itself

3. Uploaded as supplementary information.

This policy applies to all data except where public deposition would breach compliance with the protocol approved by your research ethics board. If your data cannot be made publicly available for ethical or legal reasons (e.g., public availability would compromise patient privacy), please explain your reasons by return email and your exemption request will be escalated to the editor for approval. Your exemption request will be handled independently and will not hold up the peer review process, but will need to be resolved should your manuscript be accepted for publication. One of the Editorial team will then be in touch if there are any issues.

6) Please amend your detailed Financial Disclosure statement. This is published with the article. It must therefore be completed in full sentences and contain the exact wording you wish to be published.

1) State the initials, alongside each funding source, of each author to receive each grant. For example: "This work was supported by the National Institutes of Health (####### to AM; ###### to CJ) and the National Science Foundation (###### to AM)."

2) State what role the funders took in the study. If the funders had no role in your study, please state: "The funders had no role in study design, data collection and analysis, decision to publish, or preparation of the manuscript."

3) If any authors received a salary from any of your funders, please state which authors and which funders..

If you did not receive any funding for this study, please simply state: u201cThe authors received no specific funding for this work.u201d

**Reviewers' Comments:**

Reviewer's Responses to Questions

**Key Review Criteria Required for Acceptance?**

As you describe the new analyses required for acceptance, please consider the following:

**Methods**

-Are the objectives of the study clearly articulated with a clear testable hypothesis stated?

-Is the study design appropriate to address the stated objectives?

-Is the population clearly described and appropriate for the hypothesis being tested?

-Is the sample size sufficient to ensure adequate power to address the hypothesis being tested?

-Were correct statistical analysis used to support conclusions?

-Are there concerns about ethical or regulatory requirements being met?

Reviewer #1: The WHO 2009 severity classification is mentioned later in the Introduction and results, but it should be explicitly stated at the start of the Methods section for clarity.

• The analysis includes blood transfusion requirement and hematocrit elevation as predictors of severe dengue, reporting extremely high ORs (~15-fold). However, these variables are collected during hospitalization, and may represent clinical consequences or interventions driven by severity, rather than true early predictors. This introduces high risk of reverse causation and should be carefully reframed in the discussion and conclusions.

Reviewer #2: There is a bit of a contradiction in how the study is described: it’s labeled as a ‘retrospective review,’ but the use of written consent and questionnaires suggests a more active, prospective process. It would be really helpful if the authors could clear up exactly how the study was built specifically, were patients recruited in real-time or was this a look back at a specific window? Please also clarify the timing of the interviews (for instance, were they done right at admission?) and provide a simple breakdown of which data came from the patients’ own reports versus what was pulled from their medical charts. This context is key for readers to weigh the risk of things like recall bias on symptom timing versus the objective data found in the records.

The Methods and Results sections seem to be speaking two different languages regarding diagnosis. While the Methods focus on NS1, the Results bring in IgM positivity. To make this clear for the reader, could you provide a precise definition for laboratory confirmation?

Reviewer #3: 1. The study objectives are generally clear, aiming to identify demographic, clinical, and hematological correlates of severe dengue during the 2023 Dhaka outbreak.

2. The multicenter, hospital-based observational design is appropriate for the stated objectives. However, the study is described as both cross-sectional and as involving repeated measurements, which is conceptually inconsistent. It might be more accurate if described as a retrospective observational cohort with longitudinal follow-up during hospitalization.

3. The study population is clearly described, including inclusion criteria (laboratory-confirmed dengue via NS1 or IgM positivity), setting, time frame, and hospital locations.

4. The final analytical sample is reasonably large for a hospital-based dengue study and appears sufficient for most of the primary analyses conducted.

5. Overall, the statistical methods are appropriate and well-chosen. The use of chi-squared/Fisher’s exact tests for categorical variables and parametric/non-parametric tests for continuous variables is standard. The application of generalized linear mixed-effects models to repeated measures appropriately accounts for within-patient correlation and inter-hospital variability, which is a notable strength. However, the rationale for including a requirement for blood or platelet transfusion as an adjustment variable is questionable, as this may be a consequence rather than a predictor of severity. One more thing, the handling of missing data is not described, and if present, it should be clarified.

6. The study reports ethical approval from the institutional ethical approval committee of Primeasia University, yet none of the listed authors are affiliated with Primeasia University. This raises an important ethical and procedural concern. The authors must clarify why Primeasia University served as the ethical review body; whether any author held an adjunct, visiting, or prior affiliation at Primeasia at the time of approval; and whether formal agreements existed between Primeasia University and the participating hospitals or investigators. Without clarification, this raises questions regarding ethical oversight appropriateness and institutional responsibility.

Reviewer #4: Methods are not clear for the study

**********

**Results**

-Does the analysis presented match the analysis plan?

-Are the results clearly and completely presented?

-Are the figures (Tables, Images) of sufficient quality for clarity?

Reviewer #1: • Age OR is reported as “1.77 per year”, but earlier the abstract reported OR=1.77 without specifying the unit. The per-unit vs. categorical age modeling must be clarified. ORs are reported without confidence intervals, which is required. Please add 95% CIs for all OR estimates.

• Some figure captions contain typographical issues such as “severe non-severe”, “ L!” formatting artifacts, and inconsistent units (Fahrenheit vs Celsius), which should be standardized and cleaned.

Reviewer #2: 1. There is a confusing contradiction in the data regarding BMI. The descriptive results show that severe dengue is more common in patients with a BMI of 25 or higher, but the model suggests that a higher BMI is actually protective, while the written narrative points toward lower BMI being the issue. Could you please double-check the coding and reference groups to ensure everything is aligned? It would also be helpful to clarify the exact definitions used for severe vs. non-severe outcomes.

2. Could you give us a clearer look at the big picture? It would be great to see the total number and percentage of severe dengue cases, both overall and split by site. Including confidence intervals here would really help, as it provides the necessary "anchor" for your models and plots. Without those baseline numbers, it’s a little tough for readers to fully grasp the scale and context of the results you’re showing.

3. The current analysis treats blood transfusions as a predictor of severe dengue, but since a transfusion is usually a response to a patient getting clinically worse, it likely happened after the severity was already established. This creates a "reverse causality" issue where the transfusion is more of a marker of the disease's progress than a predictor of it. Could you clarify the timing of these transfusions? It might be cleaner to remove them from your predictive models or treat them separately as a clinical outcome, keeping your "predictor" variables strictly to what was known at the time of admission.

4. The logistic regression is described as using admission values, but several covariates look like they may represent events or measurements during hospitalisation period. Please clearly define the starting time for each variable used in each model.

5. Please ensure tables specify units and denominators, include missingness, and provide full model outputs including sample size per model, CIs, and p-values

Reviewer #3: 1. Overall, the analyses presented in the results section largely align with the analysis plan described in the methods. However, result section occasionally implies predictive inference, whereas much of the analysis is, I think, associative. No big issue though.

2. This section is data-rich and generally clearly presented.

3. In general, the tables and figures are of good technical quality and enhance clarity.

Reviewer #4: Results are not clearly presented, and result has no new findings

**********

**Conclusions**

-Are the conclusions supported by the data presented?

-Are the limitations of analysis clearly described?

-Do the authors discuss how these data can be helpful to advance our understanding of the topic under study?

-Is public health relevance addressed?

Reviewer #1: There is a major inconsistency in BMI findings. The Results section states overweight individuals had a higher prevalence of severe dengue (p=0.022). Yet Table 1 (mixed-effects model) lists BMI as a strongly protective factor (OR=0.11, p<0.001). The Conclusion also emphasizes lower BMI as a risk factor. These statements contradict each other. This needs reconciliation, verification against the original data, and a corrected interpretation.

Reviewer #2: 1. Conclusions about BMI and other factors must match the corrected descriptive and multivariable results after the authors reconcile inconsistencies

2. It would be helpful to expand the limitations section to give the results more context. Specifically, please address how focusing on tertiary-care cases might introduce selection bias, and how recall bias could affect symptom timing. It’s also important to mention any missing data in the repeated labs and how clinical management decisions (like treatment timing) might have acted as a confounder. Briefly touching on these points will really help clarify the scope and reliability of the findings.

Reviewer #3: 1. The data support the conclusions. However, variables such as blood or platelet transfusion requirement have been discussed as predictors, despite being more plausibly consequences of severe disease, which weakened causal inference. The conclusion/discussion would be more robust if they more clearly distinguished associations from predictive or causal claims.

2. Are the limitations of analysis clearly described? - Yes

3. Do the authors discuss how these data can be helpful to advance our understanding of the topic under study? - Yes

4. Public health relevance is clearly acknowledged and appropriately emphasized.

Reviewer #4: yes but not clear

**********

**Editorial and Data Presentation Modifications?**

Use this section for editorial suggestions as well as relatively minor modifications of existing data that would enhance clarity. If the only modifications needed are minor and/or editorial, you may wish to recommend “Minor Revision” or “Accept”.

Reviewer #1: (No Response)

Reviewer #2: I recommend a minor revision

Reviewer #3: One minor issue in discrepancy in reported national dengue burden- in the inconsistent reporting of the total number of dengue cases during the 2023 outbreak. The manuscript variously reports figures such as over 321,000 and over 321179 cases across the abstract, introduction, and results sections.

Reviewer #4: (No Response)

**********

**Summary and General Comments**

Use this section to provide overall comments, discuss strengths/weaknesses of the study, novelty, significance, general execution and scholarship. You may also include additional comments for the author, including concerns about dual publication, research ethics, or publication ethics. If requesting major revision, please articulate the new experiments that are needed.

Reviewer #1: This manuscript presents valuable multicentre clinical data from the 2023 Dhaka dengue epidemic, a major public health crisis. The topic is highly relevant and fits the journal scope well. The dataset is substantial and the authors attempt both admission-based and longitudinal severity modeling.

However, several key issues should be addressed:

• The WHO 2009 severity classification is mentioned later in the Introduction and results, but it should be explicitly stated at the start of the Methods section for clarity.

• The analysis includes blood transfusion requirement and hematocrit elevation as predictors of severe dengue, reporting extremely high ORs (~15-fold). However, these variables are collected during hospitalization, and may represent clinical consequences or interventions driven by severity, rather than true early predictors. This introduces high risk of reverse causation and should be carefully reframed in the discussion and conclusions.

• There is a major inconsistency in BMI findings. The Results section states overweight individuals had a higher prevalence of severe dengue (p=0.022). Yet Table 1 (mixed-effects model) lists BMI as a strongly protective factor (OR=0.11, p<0.001). The Conclusion also emphasizes lower BMI as a risk factor. These statements contradict each other. This needs reconciliation, verification against the original data, and a corrected interpretation.

• Age OR is reported as “1.77 per year”, but earlier the abstract reported OR=1.77 without specifying the unit. The per-unit vs. categorical age modeling must be clarified. ORs are reported without confidence intervals, which is required. Please add 95% CIs for all OR estimates.

• Some figure captions contain typographical issues such as “severe non-severe”, “ L!” formatting artifacts, and inconsistent units (Fahrenheit vs Celsius), which should be standardized and cleaned.

• The manuscript contains several language issues that require revision (examples include: multi-canter, “were found to be higher prevalence were found”, grammar, tense inconsistency, and spacing issues). These should be corrected for publication quality.

Overall Recommendation

The manuscript is potentially acceptable after revision. The clinical insights are useful, but the paper will be significantly strengthened if the authors clearly distinguish baseline predictors vs. hospitalization markers, correct the BMI contradictions, add confidence intervals, and improve methodological transparency and language quality.

Reviewer #2: This dataset from the 2023 Dhaka epidemic is really timely and could offer great clinical and public health insights, but a few inconsistencies make the findings hard to fully follow. Right now, the study design and lab criteria feel a bit vague. There are also some contradictions in the BMI data, scaling issues with the odds ratios, and no mention of how missing data was handled. Finally, using treatments like transfusions as "predictors" is a bit circular, since they usually happen after a patient has already taken a turn for the worse. Cleaning up these points would make the paper much stronger and the results far more reliable.

Reviewer #3: This manuscript presents a timely and policy-relevant analysis of clinical and haematological correlates of severe dengue during the 2023 epidemic in Dhaka, Bangladesh. But one thing, like 2019, 2023 is a year of DENV serotype swap (from DENV-3 to DENV-2) that was one of the reasons of so much mortality and morbidity. So, author should mention this serotype swap issue or resurgence of DENV-2 in 2023 in their manuscript with proper citation.

Key strengths of the study include the multicenter design, use of standardized WHO 2009 severity criteria, rich supplementary analyses.

Weaknesses: Inconsistencies in epidemiological framing, particularly the varying reporting of national case counts (≈321,000); conceptual ambiguity in study design, described as cross-sectional despite the use of longitudinal repeated-measures analyses.

The study offers moderate novelty. Many identified risk factors are well established in a myriad of dengue literature. The primary contribution lies in context-specific epidemiological insight from the 2023 Dhaka-based outbreak.

The manuscript is well written, well structured, and supported by appropriate citations. Figures and tables are of good quality and mostly clear.

No evidence of dual publication or plagiarism is apparent. However, the ethical approval obtained from Primeasia University, despite the absence of author affiliation, requires clarification. This issue raises questions regarding institutional oversight and must be explicitly addressed to meet ethical transparency standards.

Reviewer #4: (No Response)

**********

PLOS authors have the option to publish the peer review history of their article (what does this mean?). If published, this will include your full peer review and any attached files.

If you choose “no”, your identity will remain anonymous but your review may still be made public.

**Do you want your identity to be public for this peer review?**  For information about this choice, including consent withdrawal, please see our Privacy Policy.

Reviewer #1: No

Reviewer #2: No

Reviewer #3: No

Reviewer #4: No

[NOTE: If reviewer comments were submitted as an attachment file, they will be attached to this email and accessible via the submission site. Please log into your account, locate the manuscript record, and check for the action link "View Attachments". If this link does not appear, there are no attachment files.]

**Figure resubmission:**

While revising your submission, we strongly recommend that you use PLOS’s NAAS tool (https://ngplosjournals.pagemajik.ai/artanalysis) to test your figure files. NAAS can convert your figure files to the TIFF file type and meet basic requirements (such as print size, resolution), or provide you with a report on issues that do not meet our requirements and that NAAS cannot fix.

-->

After uploading your figures to PLOS’s NAAS tool - https://ngplosjournals.pagemajik.ai/artanalysis, NAAS will process the files provided and display the results in the "Uploaded Files" section of the page as the processing is complete. If the uploaded figures meet our requirements (or NAAS is able to fix the files to meet our requirements), the figure will be marked as "fixed" above. If NAAS is unable to fix the files, a red "failed" label will appear above. When NAAS has confirmed that the figure files meet our requirements, please download the file via the download option, and include these NAAS processed figure files when submitting your revised manuscript.-->

**Reproducibility:**

To enhance the reproducibility of your results, we recommend that authors of applicable studies deposit laboratory protocols in protocols.io, where a protocol can be assigned its own identifier (DOI) such that it can be cited independently in the future. Additionally, PLOS ONE offers an option to publish peer-reviewed clinical study protocols. Read more information on sharing protocols at https://plos.org/protocols?utm_medium=editorial-email&utm_source=authorletters&utm_campaign=protocols

-->

Attachment Submitted filename: Comments_PNTD.docx

# Author comment: Author response to Decision Letter 1

**DOI:** 10.1371/journal.pntd.0013729.r002

13 Apr 2026

Attachment Submitted filename: Point-by-point Responses_PNTD-D-25-01995.docx

# Peer review: Decision Letter 1

**DOI:** 10.1371/journal.pntd.0013729.r003

**Authors:** Álvaro Acosta-Serrano, Md. Kamrujjaman

7 Jul 2026

Dear Dr. Ali,

We are pleased to inform you that your manuscript 'Clinical and haematological correlates of severe dengue using 2023 epidemic data: a multicentre tertiary hospital-based analysis from Dhaka, Bangladesh' has been provisionally accepted for publication in PLOS Neglected Tropical Diseases.

Before your manuscript can be formally accepted you will need to complete some formatting changes, which you will receive in a follow up email. A member of our team will be in touch with a set of requests.

Please note that your manuscript will not be scheduled for publication until you have made the required changes, so a swift response is appreciated.

IMPORTANT: The editorial review process is now complete. PLOS will only permit corrections to spelling, formatting or significant scientific errors from this point onwards. Requests for major changes, or any which affect the scientific understanding of your work, will cause delays to the publication date of your manuscript.

Should you, your institution's press office or the journal office choose to press release your paper, you will automatically be opted out of early publication. We ask that you notify us now if you or your institution is planning to press release the article. All press must be co-ordinated with PLOS.

Thank you again for supporting Open Access publishing; we are looking forward to publishing your work in PLOS Neglected Tropical Diseases.

Best regards,

Md. Kamrujjaman, Ph.D

Academic Editor

PLOS Neglected Tropical Diseases

Álvaro Acosta-Serrano

Section Editor

PLOS Neglected Tropical Diseases

Shaden Kamhawi

co-Editor-in-Chief

PLOS Neglected Tropical Diseases

orcid.org/0000-0003-4304-636XX

Paul Brindley

co-Editor-in-Chief

PLOS Neglected Tropical Diseases

orcid.org/0000-0003-1765-0002

***********************************************************

p.p1 {margin: 0.0px 0.0px 0.0px 0.0px; line-height: 16.0px; font: 14.0px Arial; color: #323333; -webkit-text-stroke: #323333}span.s1 {font-kerning: none

-->

Reviewer's Responses to Questions

**Key Review Criteria Required for Acceptance?**

As you describe the new analyses required for acceptance, please consider the following:

**Methods**

-Are the objectives of the study clearly articulated with a clear testable hypothesis stated?

-Is the study design appropriate to address the stated objectives?

-Is the population clearly described and appropriate for the hypothesis being tested?

-Is the sample size sufficient to ensure adequate power to address the hypothesis being tested?

-Were correct statistical analysis used to support conclusions?

-Are there concerns about ethical or regulatory requirements being met?

Reviewer #1: Yes, they addressed all the queries.

Reviewer #2: (No Response)

Reviewer #3: The study presents clearly defined objectives focused on identifying factors associated with severe dengue, and the observational hospital-based design is broadly appropriate for addressing these aims. The study population, inclusion criteria, and setting are well described and suitable for the research question, and the sample size is adequate to support the analyses performed. The statistical methods are appropriate, including the use of mixed-effects models to account for repeated measures, and prior concerns regarding the inclusion of transfusion variables have been satisfactorily addressed through appropriate model revision. Ethical considerations have also been clarified, and no major concerns remain in this regard. Overall, the authors have substantially improved the manuscript in response to reviewer comments, and although minor issues remain (e.g., precise characterization of study design and limited detail on missing data handling), these do not materially affect the validity of the findings. I recommend the manuscript for acceptance.

**********

**Results**

-Does the analysis presented match the analysis plan?

-Are the results clearly and completely presented?

-Are the figures (Tables, Images) of sufficient quality for clarity?

Reviewer #1: Results looks relevant.

Reviewer #2: (No Response)

Reviewer #3: The analyses presented are consistent with the stated analysis plan, including appropriate alignment between the described methods and reported findings. The results are clearly and comprehensively presented, with logical structuring across sections that facilitates interpretation. Tables and figures are generally of good quality and effectively support the narrative, contributing to overall clarity and readability. Minor presentation issues have been improved in the revised version and do not detract from the understanding of the results.

**********

**Conclusions**

-Are the conclusions supported by the data presented?

-Are the limitations of analysis clearly described?

-Do the authors discuss how these data can be helpful to advance our understanding of the topic under study?

-Is public health relevance addressed?

Reviewer #1: It is Okay.

Reviewer #2: (No Response)

Reviewer #3: The conclusions are supported by the data presented and are now more appropriately framed as associations rather than causal claims. The limitations of the analysis are clearly described and provide sufficient context for interpreting the findings. The authors adequately discuss how these results contribute to the broader understanding of dengue severity, particularly within the context of the 2023 outbreak in Dhaka. Public health relevance is appropriately highlighted, with clear implications for clinical management and outbreak response.

**********

**Editorial and Data Presentation Modifications?**

Use this section for editorial suggestions as well as relatively minor modifications of existing data that would enhance clarity. If the only modifications needed are minor and/or editorial, you may wish to recommend “Minor Revision” or “Accept”.

Reviewer #1: (No Response)

Reviewer #2: (No Response)

Reviewer #3: The authors have adequately addressed the major reviewer comments and improved the clarity, consistency, and methodological rigor of the manuscript. The remaining issues are minor and primarily relate to editorial clarity (e.g., precise characterization of study design and brief description of missing data handling), which do not affect the validity or interpretation of the findings. Therefore, I recommend the manuscript for acceptance.

**********

**Summary and General Comments**

Use this section to provide overall comments, discuss strengths/weaknesses of the study, novelty, significance, general execution and scholarship. You may also include additional comments for the author, including concerns about dual publication, research ethics, or publication ethics. If requesting major revision, please articulate the new experiments that are needed.

Reviewer #1: Authors addressed all the comments.

Reviewer #2: (No Response)

Reviewer #3: This manuscript presents a timely and relevant analysis of factors associated with severe dengue during the 2023 outbreak in Dhaka, with key strengths including its multicentre design, use of standardized WHO criteria, and integration of clinical and longitudinal data. The authors have adequately addressed prior reviewer concerns, particularly regarding methodological clarity, handling of transfusion variables, and ethical approval, and the manuscript is now clearer and more consistent, with findings appropriately interpreted as associations. While minor issues remain, such as some imprecision in study design wording and limited detail on missing data handling, these do not materially affect the validity or interpretation of the results. Overall, the study provides useful context-specific insights with clear public health relevance and is suitable for publication.

**********

PLOS authors have the option to publish the peer review history of their article (what does this mean?). If published, this will include your full peer review and any attached files.

If you choose “no”, your identity will remain anonymous but your review may still be made public.

**Do you want your identity to be public for this peer review?**  For information about this choice, including consent withdrawal, please see our Privacy Policy.

Reviewer #1: No

Reviewer #2: No

Reviewer #3: No

# Editor report: Acceptance letter

**DOI:** 10.1371/journal.pntd.0013729.r004

**Authors:** Álvaro Acosta-Serrano, Md. Kamrujjaman

Dear Dr. Ali,

We are delighted to inform you that your manuscript, "Clinical and haematological correlates of severe dengue using 2023 epidemic data: a multicentre tertiary hospital-based analysis from Dhaka, Bangladesh," has been formally accepted for publication in PLOS Neglected Tropical Diseases.

We have now passed your article onto the PLOS Production Department who will complete the rest of the publication process. All authors will receive a confirmation email upon publication.

The corresponding author will soon be receiving a typeset proof for review, to ensure errors have not been introduced during production. Please review the PDF proof of your manuscript carefully, as this is the last chance to correct any scientific or type-setting errors. Please note that major changes, or those which affect the scientific understanding of the work, will likely cause delays to the publication date of your manuscript. Note: Proofs for Front Matter articles (Editorial, Viewpoint, Symposium, Review, etc...) are generated on a different schedule and may not be made available as quickly.

Soon after your final files are uploaded, the early version of your manuscript will be published online unless you opted out of this process. The date of the early version will be your article's publication date. The final article will be published to the same URL, and all versions of the paper will be accessible to readers.

For Research Articles, you will receive an invoice from PLOS for your publication fee after your manuscript has reached the completed accept phase. If you receive an email requesting payment before acceptance or for any other service, this may be a phishing scheme. Learn how to identify phishing emails and protect your accounts at https://explore.plos.org/phishing.

Thank you again for supporting open-access publishing; we are looking forward to publishing your work in PLOS Neglected Tropical Diseases.

Best regards,

Shaden Kamhawi

co-Editor-in-Chief

PLOS Neglected Tropical Diseases

Paul Brindley

co-Editor-in-Chief

PLOS Neglected Tropical Diseases
